# Revolutionizing Radiology with Natural Language Processing and Chatbot Technologies: A Narrative Umbrella Review on Current Trends and Future Directions

**DOI:** 10.3390/jcm13237337

**Published:** 2024-12-02

**Authors:** Andrea Lastrucci, Yannick Wandael, Angelo Barra, Renzo Ricci, Antonia Pirrera, Graziano Lepri, Rosario Alfio Gulino, Vittorio Miele, Daniele Giansanti

**Affiliations:** 1 Department of Allied Health Professions, Azienda Ospedaliero-Universitaria Careggi, 50134 Florence, Italy; andrea.lastrucci@unifi.it (A.L.); wandaely@aou-careggi.toscana.it (Y.W.); barraa@aou-careggi.toscana.it (A.B.); riccire@aou-careggi.toscana.it (R.R.); 2 Centro TISP, ISS Via Regina Elena 299, 00161 Rome, Italy; antonia.pirrera@iss.it; 3 Azienda Unità Sanitaria Locale Umbria 1, Via Guerriero Guerra 21, 06127 Perugia, Italy; graziano.lepri@uslumbria1.it; 4 Facoltà di Ingegneria, Università di Tor Vergata, Via del Politecnico, 1, 00133 Rome, Italy; gulino@disp.uniroma2.it; 5 Department of Experimental Clinical and Biomedical Sciences, University of Florence, 50134 Florence, Italy; vmiele@sirm.org; 6 Department of Radiology, Careggi University Hospital, 50134 Florence, Italy

**Keywords:** radiology, natural language model, natural language processing, chatbot, ChatGPT

## Abstract

The application of chatbots and NLP in radiology is an emerging field, currently characterized by a growing body of research. An umbrella review has been proposed utilizing a standardized checklist and quality control procedure for including scientific papers. This review explores the early developments and potential future impact of these technologies in radiology. The current literature, comprising 15 systematic reviews, highlights potentialities, opportunities, areas needing improvements, and recommendations. This umbrella review offers a comprehensive overview of the current landscape of natural language processing (NLP) and natural language models (NLMs), including chatbots, in healthcare. These technologies show potential for improving clinical decision-making, patient engagement, and communication across various medical fields. However, significant challenges remain, particularly the lack of standardized protocols, which raises concerns about the reliability and consistency of these tools in different clinical contexts. Without uniform guidelines, variability in outcomes may hinder the broader adoption of NLP/NLM technologies by healthcare providers. Moreover, the limited research on how these technologies intersect with medical devices (MDs) is a notable gap in the literature. Future research must address these challenges to fully realize the potential of NLP/NLM applications in healthcare. Key future research directions include the development of standardized protocols to ensure the consistent and safe deployment of NLP/NLM tools, particularly in high-stake areas like radiology. Investigating the integration of these technologies with MD workflows will be crucial to enhance clinical decision-making and patient care. Ethical concerns, such as data privacy, informed consent, and algorithmic bias, must also be explored to ensure responsible use in clinical settings. Longitudinal studies are needed to evaluate the long-term impact of these technologies on patient outcomes, while interdisciplinary collaboration between healthcare professionals, data scientists, and ethicists is essential for driving innovation in an ethically sound manner. Addressing these areas will advance the application of NLP/NLM technologies and improve patient care in this emerging field.

## 1. Introduction

### 1.1. Background

Traditional radiology, which relied on film-based imaging and manual interpretation, was the standard for many decades. The process was often labor-intensive, requiring physical handling of films and a slower pace of analysis. Radiologists would examine these films under specific lighting conditions, with limited ability to quickly share or store images for collaborative analysis. While it was a significant step forward in medical imaging, traditional radiology had its limitations, including issues with image quality, difficulty in sharing results, and challenges in maintaining consistent standards across different institutions. However, the transition to digital radiology in the late 20th and early 21st centuries dramatically changed this landscape.

Radiology has evolved significantly due to standardization processes over the past millennium. The introduction of DICOM (Digital Imaging and Communications in Medicine) [1,2,3] and the integration of Radiology Information Systems (RISs) with Picture Archiving and Communication Systems (PACSs) [4,5] were key milestones [6]. Initially, these advances focused on traditional radiology modalities like X-rays and CT scans. Over time, they expanded to include a wide range of diagnostic imaging techniques [3].

DICOM [3] and RISs [4] have improved the consistency and interoperability of imaging systems, enabling better integration across modalities. This includes both ionizing radiation methods (X-rays) and non-ionizing techniques (ultrasound, MRI). While “digital radiology” is sometimes used broadly, it typically refers to DICOM-compliant digital technologies in radiology.

On the other hand, integrating tissue and cellular imaging with digital health systems has been slower. Digital pathology, covering histology and cytology, adopted digital technologies more gradually, partly due to the delayed introduction of DICOM standards for Whole-Slide Imaging (WSI) [7,8]. Unlike diagnostic imaging, DICOM standards for WSI were released later, slowing integration.

The rapid adoption of digital radiology has paved the way for the introduction of AI in radiology [9]. Building on these advancements, the integration of artificial intelligence (AI) into radiology has become a natural next step in this evolution [9,10]. As digital systems provided the infrastructure for handling large volumes of imaging data, AI technologies began to emerge, capable of analyzing these datasets more rapidly and with greater accuracy than human radiologists alone. AI’s ability to detect patterns, assist in diagnostics, and even predict future medical conditions holds tremendous promise, ushering in a new era of medical imaging. The combination of digital imaging and AI is expected to further revolutionize radiology, improving both patient outcomes and workflow efficiency, offering unprecedented opportunities for innovation in diagnostic imaging. Since the COVID-19 pandemic, AI research in radiology has surged, with numerous publications appearing post-2020. A simple PubMed search shows a significant increase in AI-related research in radiology. For more details, see the growing publications on AI and radiology after 2020 [11]. The COVID-19 pandemic accelerated the adoption of artificial intelligence (AI) in radiology due to the urgent need for predictive diagnostics, particularly in medical imaging [12]. This urgency spurred unprecedented collaboration among diverse disciplines, including radiology, medical physics, imaging science, computer science, and informatics, to develop innovative AI solutions [13,14]. The rapid convergence of these fields revealed challenges stemming from education [15,16] and career transitions between domains, such as adapting AI for non-imaging data to imaging data, transitioning from clinical imaging roles to AI-focused research, and applying general AI methods to COVID-19-specific imaging. Lessons learned from these transitions highlight the need to address discipline-specific complexities to improve the integration and effectiveness of AI. Today, AI applications in radiology extend well beyond emergency responses, transforming the field through innovations like automated detection systems for breast cancer in mammography, segmentation of brain tumors in MRI scans, and enhanced analysis of liver fibrosis in ultrasound imaging [17]. AI-powered algorithms are also streamlining workflows, improving reporting efficiency, and reducing diagnostic errors. Furthermore, predictive models are being employed to assess disease progression, assist in treatment planning, and enable precision medicine approaches. These developments illustrate AI’s growing impact on radiology, not only as a tool for addressing specific imaging challenges but also as a catalyst for advancing personalized and efficient healthcare delivery.

AI’s enhanced capabilities are expected to revolutionize diagnostic imaging by improving accuracy, efficiency, and predictive analytics, offering new opportunities for innovation and patient care. These advancements in AI highlight its potential to elevate radiology’s effectiveness in diagnosing complex conditions and streamlining workflows.

Building on this progress, another pivotal transformation in healthcare involves the emergence of large language models (LLMs) [18] and AI-based chatbots [19]. While diagnostic AI primarily focuses on image analysis and predictive modeling, LLMs and AI chatbots aim to improve how healthcare professionals and patients interact with medical information. These tools, grounded in natural language processing, enable efficient data synthesis, report generation, and conversational interfaces, bridging the gap between data-driven diagnostics and user-centric communication solutions.

This complementary evolution of AI technologies reflects a broader trend toward integrating innovative tools across healthcare domains, enhancing both clinical decision-making and patient engagement.

Large language models (LLMs) are advanced AI algorithms trained on large text corpora, which enables them to understand and generate coherent, contextually relevant language. This capability allows LLMs to handle complex tasks, such as content analysis, report generation, and synthesizing diverse information. They are particularly useful for medical applications, where they can assist in generating comprehensive imaging reports and processing intricate medical data. LLMs are designed to process a wide range of topics with deep language comprehension, making them effective for diverse uses in healthcare [18].

In contrast, AI-based chatbots are systems designed to interact with users through natural language. They assist with a variety of tasks, such as answering patient questions, appointment scheduling, and managing administrative functions, typically following predefined scripts or interactions. While both LLMs and chatbots leverage AI to process and generate text, chatbots are primarily optimized for interactive, structured conversations with users. They differ from LLMs in that they are specialized for specific, task-oriented communications rather than broad content generation [19].

The key distinction between these two technologies is crucial for their application in healthcare. LLMs excel at understanding and generating complex information across multiple domains, which allows them to support detailed reporting and medical documentation. Chatbots, however, are designed to facilitate real-time, interactive communication, helping to streamline tasks such as patient triage or answering frequently asked questions in radiology settings. These technologies can complement one another in enhancing both administrative workflows and clinical decision-making in healthcare environments [18,19].

Historically, chatbots were used in healthcare before the widespread adoption of AI. Early systems included [20] the following:

Automated Response Systems (ARSs): these were basic, pre-programmed systems designed to handle simple queries.

Virtual Assistants (VAs): more sophisticated tools capable of assisting with a broader array of tasks.

Agent-Based Systems (ABSs): these chatbots were used for more technical or specific tasks, such as those in medical imaging, providing the early foundations for the AI-driven systems used today.

These earlier systems laid the groundwork for the more advanced AI-based chatbots now being integrated into radiology, improving both operational efficiency and patient care through automation and support.

Chatbots have been in radiology since 1991, as shown by a PubMed search with the term “chatbot in radiology: historical view” [21]. Chatbots in radiology have evolved significantly, shifting from simple administrative tools to advanced systems that directly support radiologists in clinical and research workflows. Initially, chatbots were used for patient communication, such as appointment scheduling and answering basic queries about imaging procedures. Over time, their capabilities expanded to include clinical applications like interpreting imaging protocols, providing decision support, and assisting in diagnostic workflows.

Key milestones in their evolution include the following:

- Workflow optimization (pre-2020) [22]: Chatbots were integrated into healthcare (also in radiology practices) to streamline administrative tasks and improve communication between patients and providers. They were instrumental in improving efficiency and reducing radiologist workloads by addressing repetitive tasks.
- COVID-19 Applications (2020–2021) [23]: During the pandemic, chatbots supported triage and managed the influx of imaging requests. They facilitated remote consultations and helped explain imaging findings to patients, playing a crucial role in maintaining healthcare access.
- LLM-Enhanced Systems (post-2021) [24,25]: The integration of large language models (LLMs) has enabled chatbots to provide sophisticated support in interpreting imaging data, suggesting differential diagnoses, and even assisting in educational initiatives for radiologists and trainees.

With technological advancements, “chatbot” is now the predominant term.

Models like ChatGPT [26], based on LLMs, have appeared in PubMed since 2022, with a marked increase in publications from 2022 [27]. The entry of ChatGPT into PubMed, particularly in radiology, became noticeable from 2023 onward [28].

### 1.2. Objectives and Rationale for Investigating LLMs and AI-Based Chatbots in Radiology

**The following is as observed:**

The integration of large language models (LLMs) and AI-based chatbots into radiology has been a transformative process. Initially, chatbots in healthcare including radiology were used primarily for administrative tasks such as appointment scheduling and answering basic patient queries. However, as AI technologies evolved, so did the role of chatbots in radiology. They began supporting radiologists in clinical workflows by optimizing administrative processes and streamlining communication, which in turn allowed radiologists to focus more on diagnostic tasks.

LLMs, which specialize in complex language tasks, and AI-based chatbots, which excel in interactive, context-specific communication, are now significantly shaping the field. Chatbots have advanced to assist with clinical decision-making, interpreting imaging protocols, and even aiding in the diagnostic process. Notably, during the COVID-19 pandemic, these systems played a pivotal role in managing radiological workflows, offering triage support, and facilitating remote consultations.

The adoption of these technologies in radiology has followed a clear progression. From simple virtual assistants to sophisticated AI models, chatbots are now integrated into diagnostic and educational practices. LLMs, in particular, have enabled chatbots to provide more advanced support, offering differential diagnoses and assisting in radiologist training. This transition has been fueled by the growing need for accuracy, efficiency, and predictive analytics in medical imaging.

**In light of this, the following is observed:**

Our *general objective* is to investigate, through an umbrella review of systematic reviews, the current state of introduction and integration of large language models (LLMs) and AI-based chatbots in radiology. The sub-aims are as follows:

*Evaluate contributions*: Examine and categorize the ways in which LLMs and chatbots are contributing to the field of radiology. This will involve analyzing emerging themes and assessing their overall impact on radiological practices and processes.

*Explore opportunities and challenges*: Identify and explore the opportunities presented by these technologies in radiology, as well as the challenges that need to be addressed. This includes understanding both the potential benefits and the obstacles to effective implementation.

Recommendations will be developed and elaborated in the discussion, based on the findings from this review. These recommendations will aim to guide the future integration and utilization of LLMs and chatbots in radiology, with a focus on enhancing effectiveness and encouraging adoption.

## 2. Methods

### 2.1. Search Strategies and Used Narrative Checklist

A narrative review of reviews was conducted, focusing on the field of the intersection of radiology with NLP and chatbots. A standardized checklist for narrative reviews, the ANDJ Narrative Checklist, is available online. A link to the checklist [29] was used. The *ANDJ Narrative Review Checklist* [29] is a tool designed to assist in conducting high-quality narrative reviews. It guides researchers and authors through the process of writing a narrative review, ensuring that all relevant aspects are considered and the review is clear, comprehensive, and rigorous. Starting with the title, Checklist Item 1 emphasizes the importance of explicitly identifying the report as a narrative review. This clarity helps readers immediately understand the nature of the article, setting the stage for the content to follow. A well-crafted title is the first step in ensuring the review’s purpose is clear from the outset.

The abstract, as described in Checklist Item 2, should provide an unstructured summary of the review, covering the background; objectives; a brief synthesis of the review’s content; and the implications for future research, clinical practice, or policy. This succinct overview is vital for quickly communicating the significance of the review to its audience, offering them a glimpse of what the article will deliver.

In the introduction, Checklist Item 3 directs authors to explain the rationale for the review in the context of existing knowledge. This section is important for highlighting the topic’s relevance, establishing the review’s significance, and underscoring the gap in the literature that the review aims to address. Additionally, Checklist Item 4 recommends that authors specify the key questions guiding the review, ensuring a clear focus throughout the article.

Moving on to the methods, Checklist Item 5 advises authors to detail the research selection process thoroughly. This should include the years of literature considered, language, publication status, study design, and the databases used for the search. This transparency is essential to allow readers to understand how the studies were identified and selected, thereby enhancing the credibility of the review.

The discussion section, covered in Checklist Item 6, is where authors are expected to engage deeply with the research they reviewed. This discussion should highlight the fundamental findings, address the limitations and quality of the studies examined, and identify any gaps in the literature. Importantly, it should emphasize the need for future research to further explore these gaps or build upon the current understanding.

Finally, in the summary, as outlined in Checklist Item 7, the narrative review should provide an overall interpretation, contextualizing the findings for health professionals, policy makers, and researchers. This section should also direct attention to future research directions, suggesting how the current review fits into the broader landscape and contributes to the development of the field.

By following the ANDJ Narrative Review Checklist, authors ensure their reviews are comprehensive, well organized, and impactful, providing valuable insights for advancing knowledge, improving clinical practice, and informing policy development.

The search was based on targeted searches on Google scholar, Pubmed, and Scopus.

The composite key was “((chatbot[Title/Abstract]) OR (NLP[Title/Abstract]) OR (chatgpt[Title/Abstract]) OR(natural language processing[Title/Abstract]) OR (natural language model[Title/Abstract])) AND (radiology [Title/Abstract])”, with the search also repeated in [full text] in addition to [Title/abstract].

### 2.2. Assessment Criteria for Inclusion

To ensure a comprehensive and high-quality narrative review, each selected study was evaluated according to the following criteria [30]:

Clarity of Rationale (N1): This criterion assesses whether the study clearly explains the reason for its investigation. The rationale should define the research problem, emphasize its significance, and justify the necessity of the study. A well-defined rationale establishes context and provides a strong justification for the research effort. For example, studies should identify gaps in existing knowledge or practice and demonstrate the relevance of the research to algorithms and ethics.

Design Appropriateness (N2): This criterion evaluates whether the study design is appropriate for answering the research question or hypothesis. The design should align with the study’s objectives and scope. An appropriate design includes selecting the correct methodology, sample size, and data collection methods.

Methodological Clarity (N3): This criterion refers to how clearly the study’s methods are described and whether they are replicable. This includes the transparency of data collection, analysis, and interpretation procedures. The study should offer detailed information on how data were gathered, the tools and techniques used, and how the analysis was performed, ensuring that the study can be reproduced or critiqued based on the methodology provided.

Result Presentation (N4): This criterion assesses how clearly and effectively the study presents its results. The findings should be well organized, accurately reported, and appropriately interpreted. The presentation should include relevant tables, figures, and statistical analyses that support the conclusions drawn.

Justification of Conclusions (N5): This criterion evaluates whether the study’s conclusions are supported by its results. The study should establish a logical connection between the data and the conclusions, discuss the implications of the findings, address limitations, and suggest future research areas. Justifying the conclusions ensures that the outcomes are valid and based on solid evidence.

Disclosure of Conflicts of Interest (N6): Transparency regarding conflicts of interest is essential for evaluating the impartiality of the study. This criterion checks whether the authors disclose any financial, professional, or personal interests that could influence the research. Full disclosure ensures the objectivity of the study and prevents external biases from affecting the results.

The selected studies were evaluated based on the five parameters (N1–N5), each scored on a scale from 1 (minimum) to 5 (maximum), and one binary parameter (N6) assessing the Disclosure Of Conflicts Of Interest (Yes/No). To be included in this review, studies had to meet the following criteria: parameter N6 must be “Yes”, and the parameters N1–N5 must each score above 3.

### 2.3. Assessment Process

Each study was reviewed by two initial assessors ([DG], [AL]). These assessors were responsible for evaluating each study based on its focus on algorethics, using the criteria defined in the previous section. Each criterion—Clarity of Rationale, Design Appropriateness, Methodological Clarity, Result Presentation, Justification of Conclusions, and Disclosure of Conflicts of Interest—was scored on a predefined scale to quantitatively measure the quality and relevance of each study.

The primary assessors independently reviewed the studies and assigned scores for each parameter, ensuring consistent evaluation across all studies. This dual-assessment approach was intended to enhance the reliability of this review by capturing multiple perspectives and reducing potential individual biases.

If the two primary assessors disagreed on the scores or the inclusion of a study, a third assessor ([AP] or [GL]) was brought in to resolve the disagreement. This third-party assessment was crucial for ensuring fairness and transparency in the decision-making process, as it helped to balance differing opinions and add an extra layer of scrutiny.

The multi-assessor approach was designed to minimize bias and ensure a thorough, balanced evaluation of the literature. By incorporating diverse perspectives and implementing a structured method for resolving disagreements, this review aimed to offer a comprehensive and objective assessment of the studies’ relevance to algorethics in the health domain.

### 2.4. Managing Bias in This Narrative Review

To ensure this narrative review remained objective and rigorous, several strategies were used to manage and minimize bias throughout the assessment process. These strategies include the following:

Diverse Assessors: Each study was reviewed by two primary assessors ([DG] and [AL]) from different backgrounds, ensuring a broad range of perspectives and minimizing individual biases in the evaluation process.

Clear Assessment Criteria: The evaluation was based on predefined parameters—Clarity of Rationale, Design Appropriateness, Methodological Clarity, Result Presentation, Justification of Conclusions, and Disclosure of Conflicts of Interest. Using standardized criteria helped to reduce the risk of subjective interpretation and ensured consistency across studies.

Scoring system: Each parameter was scored on a scale from 1 to 5, and conflicts of interest were assessed on a binary Yes/No basis. This quantifiable approach allowed for a consistent and transparent evaluation across studies.

Independent Review: Primary assessors independently reviewed the studies without prior consultation. This approach minimized the influence of groupthink and ensured that judgments were based solely on the studies’ merits and predefined criteria.

Dispute Resolution: In cases of disagreement between the two primary assessors, a third assessor ([AP] or [GL]) was involved to resolve the conflict. This third-party adjudication provided an impartial perspective, ensuring fair resolution of differences and adding an extra layer of scrutiny to the review process.

Structured Mechanism for Disagreements: Disagreements were resolved using a formalized process. The third assessor reviewed the initial evaluations and provided a reasoned judgment to reconcile differences, ensuring that conflicts were addressed systematically.

Transparency: A standardized checklist and a clear scoring system ensured transparency in the assessment process. By documenting the criteria and rationale behind the scores, the review process was made transparent, reducing the risk of undisclosed biases.

By employing these strategies, this review aimed to provide a thorough and balanced evaluation of the literature, minimizing bias and enhancing the objectivity and reliability of the review process.

### 2.5. Selected Studies

**Figure 1:**
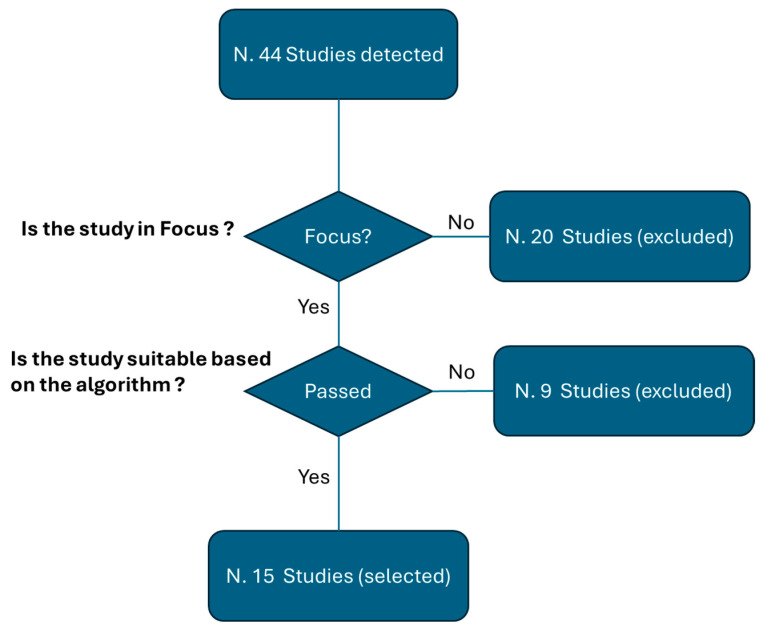
Flow of study selection.

At the end of the selection process, the final set of studies was identified. Figure 1 illustrates the steps involved, showing that the initial search resulted in 44 studies. From these, 20 studies were excluded due to their lack of a direct focus. Following the evaluation process outlined in Section 2.2 and Section 2.3, 15 studies were ultimately selected for inclusion in the review.

## 3. Results

The results have been systematically organized into two four subsections.

Section 3.1 synthesizes findings from systematic reviews on NLP and AI applications in radiology, analyzing key developments, methodologies, and challenges identified in studies from 2016 onward. This is conducted both specifically for each study (with attention to methodological aspects, focus, results, and practical and ethical implications) and globally, providing an analysis of the field’s evolution. It addresses the practical and ethical implications of integrating these technologies into clinical workflows, identifies critical barriers such as data sharing and system validation, and proposes potential solutions for overcoming them. By examining the evolving role of NLP and chatbots in radiology, this section reconstructs the marked evolution of the field as reflected in the studies and serves as a narrative bridge between the analytical trends explored in the following sections, while also responding to the specific sub-objectives of this umbrella review.

Section 3.2 examines the prevailing trends observed in the studies within this field.

Section 3.3 addresses the sub-aims of the review, focusing on the following key areas:

- **3.3.1 Response to specific sub-aim 1:** Evaluate contributions. This section examines and categorizes the contributions of LLMs and chatbots in radiology. It involves analyzing emerging themes and evaluating the overall impact these technologies have on radiological practices and processes.
- **3.3.2 Response to specific sub-aim 2:** Explore opportunities and challenges. This section identifies and explores the opportunities that LLMs and chatbots present in radiology, as well as the challenges they introduce. The analysis includes both the potential benefits and the obstacles to effective implementation.

Section 3.4 reports a synoptic diagram resuming the highlight reporting in tables/figures.

Overall, this organization ensures a systematic and logical presentation of findings, aligned with this umbrella review’s objectives:

**Clear subdivision (3.1–3.4):** Each subsection has a distinct focus, progressing from a detailed synthesis (3.1) to broader trend analysis (3.2), targeted sub-aims (3.3), and a visual synthesis (3.4). This structured approach enhances clarity and coherence.

**Section 3.1:** It synthesizes findings from systematic reviews, analyzing key developments, methodologies, and challenges while tracing the field’s evolution and addressing practical and ethical implications. Emerging trends are introduced narratively through qualitative analysis.

**Section 3.2, Section 3.3 and Section 3.4:** Section 3.2 deepens the analysis of trends introduced narratively in Section 3.1 through graphical and statistical methods for a more robust perspective. Section 3.3 evaluates the contributions and challenges of LLMs and chatbots, building on the evidence presented in Section 3.1. Section 3.4 concludes with a visual summary, providing a comprehensive and accessible overview.

### 3.1. Synthesizing Findings, Critical Perspectives, and Future Directions in NLP and AI Applications in Radiology

This section explores the evolution of natural language processing (NLP) and chatbot technologies in radiology, based on systematic reviews spanning from 2016 to the present. Drawing upon studies [31,32,33,34,35,36,37,38,39,40,41,42,43,44,45], we analyze the methodologies, findings, and practical implications of these technologies in the radiological domain.

The section begins with an overview of the development of NLP tools and chatbot applications, highlighting key advancements and their integration into radiological workflows. It examines the methodologies used in the selected studies, providing insights into the technical frameworks, evaluation metrics, and performance standards applied in assessing these systems, and particular attention is devoted to the practical and ethical implications.

Key challenges are identified, including the issues of data sharing, system validation, and the integration of NLP tools into clinical practice. These difficulties are discussed in a structured manner, with potential solutions, such as improved data-sharing protocols and standardized validation frameworks, being suggested to address the limitations of current technologies.

The ethical implications of NLP and chatbot technologies are also a focal point, with attention given to data privacy concerns, algorithmic bias, and the impact these issues may have on clinical decision-making and patient care. A critical discussion is provided on the need for ethical guidelines that ensure transparency and fairness in the development and deployment of these tools.

Finally, the section outlines future research directions, emphasizing the need for more specific, targeted studies that address the practical challenges and ethical concerns identified. Suggestions for further advancements in NLP and chatbot technologies are proposed, ensuring that these tools can be deployed safely and effectively in radiology, with a focus on enhancing both patient outcomes and healthcare efficiency.

#### 3.1.1. Progression of Studies on NLP and Chatbot Applications in Radiology: Key Developments and Implications

The field of natural language processing (NLP), including chatbot applications, in radiology has witnessed remarkable progress, starting with foundational reviews and culminating in real-world applications that promise to transform clinical workflows. A critical turning point occurred in 2016, when Pons et al. [39] published the first comprehensive systematic review of NLP in radiology, which provided a solid foundation for future developments. This review was pivotal in highlighting the potential of NLP technologies to extract structured data from the vast amounts of unstructured, free-text radiology reports stored in electronic health records. The 2016 study by Pons et al. [39] examined 67 publications on NLP in radiology, focusing on methods to automatically extract meaningful data from radiology reports. The study emphasized both the promise of NLP to improve clinical decision-making, enhance research, and streamline workflows, as well as the challenges associated with its implementation. These challenges included issues of accuracy, adaptability to various reporting formats, and the need for standardization in NLP methods. Despite these hurdles, the review set the stage for the growing interest in NLP in the years to follow, signaling its transformative potential in radiology. Following Pons et al.’s groundwork, researchers began exploring the application of NLP in broader aspects of radiology. From 2017 to 2019, the body of the literature on NLP grew substantially, with studies focusing on improving information extraction and leveraging deep learning techniques for more complex analysis. During this period, Blackley et al. [44] conducted another systematic review, this time exploring speech recognition (SR) technology in clinical documentation, which included radiology. Their review demonstrated that while speech recognition was advancing, challenges around accuracy and integration into clinical systems remained significant. By 2020, NLP in radiology began to shift towards more advanced techniques, particularly deep learning models. For instance, Sorin et al. [43] applied deep learning-based NLP models to analyze radiology reports for specific conditions, such as pulmonary embolism. The increasing sophistication of neural networks and convolutional neural networks (CNNs) marked a leap in the ability to analyze medical data more efficiently and accurately. This era marked the start of more complex NLP applications, with a shift from rule-based methods to data-driven, machine learning models that were capable of learning from vast datasets and improving over time. As NLP technologies in radiology became more advanced, the importance of standardization and external validation came to the forefront. In 2021, Davidson et al. [41] published a systematic review that identified key gaps in the NLP research landscape, particularly around the lack of standardized reporting and the need for external validation of NLP models before they could be implemented in real-world clinical settings. This review underscored the variability in NLP model performance and highlighted the necessity of consistent protocols and metrics for evaluating NLP technologies across studies. In recent years, chatbots, as a subset of NLP applications, have also emerged in the clinical setting. These AI-driven systems have been explored for improving structured radiology reporting, offering assistance in interpreting and generating reports based on radiological data. Notable systematic reviews, such as those by Sacoransky et al. [33], Keshavarz et al. [34], and Temperley et al. [35], have addressed the evolving role of chatbots in radiology, highlighting their potential for enhancing clinical workflows while also discussing performance limitations and challenges regarding integration into existing systems. In 2024, Klug et al. [31] explored the potential of NLP tools across the patient journey, from admission to discharge, presenting a thorough systematic review that underscored how NLP can optimize patient management, especially in terms of documentation and discharge planning. Younis et al. [32] conducted another comprehensive review on AI in healthcare, detailing its applications and challenges, including the use of NLP for data extraction and the broader context of its adoption in medicine. Their analysis suggests that while NLP can be a valuable tool in healthcare, it faces challenges related to data privacy, scalability, and ethical concerns. Furthermore, in 2024, a systematic review by Keshavarz et al. [34] on ChatGPT in radiology emphasized its potential to aid in structured radiology reporting, particularly in generating initial draft reports, but also pointed out the model’s limitations in dealing with complex cases. Similarly, in the same year, the study by Temperley et al. [35] highlighted the growing role of ChatGPT in radiology, stressing its future potential to assist radiologists in report writing and the challenges that lie in the integration of such technologies into routine clinical practice. In the area of breast imaging, Diab et al. [37] reviewed NLP applications for breast cancer detection, noting the improvements in diagnostic accuracy and workflow efficiency that NLP tools can bring, particularly for radiology reports. A similar the review by Saha et al. [38] addressed the specific use of NLP for breast cancer, focusing on how structured data can be extracted from reports to improve diagnostic consistency and support clinical decision-making. Despite the rapid growth in NLP applications, challenges remain. The recent studies by Sacoransky et al. [33] and Keshavarz et al. [34] underscore the persistent issues of model reliability, data privacy concerns, and the need for validation in real-world clinical environments. These reviews indicate that while NLP, including chatbot applications, holds significant promise, a cautious approach is necessary when implementing these technologies in healthcare settings. From 2022 onward, the focus shifted to real-world deployment, ethical considerations, and overcoming systemic barriers. Recent studies have started emphasizing the importance of ensuring that NLP technologies are not only effective but also transparent, accountable, and ethical in their use. Issues such as patient data privacy, algorithmic transparency, and bias in training data have become central to the discourse on NLP in healthcare. Researchers and practitioners have increasingly called for interdisciplinary collaboration to ensure the responsible deployment of NLP in clinical environments, with an emphasis on ethics and regulatory oversight. The journey from 2016 to the present has been marked by continuous progress, but challenges remain. The early systematic reviews, starting with Pons et al. [39], set the stage for exploring the potential of NLP in radiology, revealing both the promise and the hurdles of these technologies. As the field continues to evolve, future research will likely focus on improving model accuracy, ensuring data privacy, and standardizing reporting practices to enable the broader adoption of NLP tools, including chatbots, in clinical settings. NLP, including chatbot applications, is expected to play an increasingly significant role in automating data extraction, enhancing diagnostic accuracy, and improving workflow efficiency in radiology. However, for this vision to be fully realized, continued advancements in technology, collaboration across disciplines, and a commitment to ethical standards will be essential.

#### 3.1.2. Detailed Analysis of Studies on NLP and Chatbot Applications in Radiology: Focus, Methodologies, Findings, and Practical and Ethical Implications

For each study, an analysis is provided focusing on the following aspects:

Focus: this section outlines the primary subject of the study, specifying the central issue or technology being investigated.

Methodology: the methods employed in the study are described, including the research design, tools, and data collection and analysis techniques.

Results: the main findings of the study are summarized, highlighting the conclusions drawn and their contribution to the field.

Ethical and practical implications: the ethical and practical consequences of the study’s findings are discussed, including issues such as data privacy, fairness, and potential real-world applications.

Each study is analyzed through these dimensions, offering a comprehensive overview of its objectives, approach, outcomes, and broader implications.

Klug et al. (2024) [31]. From admission to discharge: a systematic review of clinical natural language processing along the patient journey.

Focus: The study investigates the application of natural language processing (NLP) in healthcare, specifically within the hospital environment, where a wealth of structured and unstructured medical text is generated. It aims to classify existing NLP approaches and map them onto the stages of the patient journey, highlighting both well-researched areas like diagnosis, admission, and discharge, and neglected ones, such as treatment, billing, and aftercare. Central to the research is understanding how NLP can improve clinical decision-making and operational efficiency while addressing key challenges such as bias and model explainability.

Methodology: A systematic review of the literature was conducted, using a novel framework that aligns research findings with the patient journey in hospitals. This approach evaluates not only the stages where medical documents are produced and used but also the types of datasets, languages, and model architectures involved. The methodology extends to identifying barriers to implementation, particularly focusing on ethical concerns such as fairness and transparency, which are essential for the successful adoption of NLP in clinical practice.

Results: The study reveals that while NLP has been extensively applied to certain patient journey stages, many crucial phases, such as aftercare and smart home integration, remain underexplored. Most current models are trained on a limited subset of documents, like radiology reports and discharge summaries, ignoring the broader range of medical texts available. This focus represents a missed opportunity to utilize NLP more comprehensively, particularly in areas that could enhance continuity of care and long-term patient outcomes.

Practical and ethical implications: The findings underscore the need to expand NLP applications to cover all stages of the patient journey. Integrating NLP into neglected areas, such as aftercare and billing, could improve care coordination and reduce administrative burdens, ultimately fostering innovation in hospital workflows. Ethically, the research highlights the importance of addressing biases inherent in training datasets and enhancing model transparency to build trust among healthcare providers and patients. These efforts are critical for ensuring the equitable and responsible deployment of NLP technologies, aligning with broader goals of system-level innovation in healthcare.

Younis et al. (2024) [32]. A Systematic Review and Meta-Analysis of Artificial Intelligence Tools in Medicine and Healthcare: Applications, Considerations, Limitations, Motivation and Challenges.

Focus: The study explores the transformative role of large language models (LLMs), such as ChatGPT, in healthcare. It emphasizes their potential to enhance medical practices, improve patient care, and support interactions between healthcare professionals, patients, and data. Specific applications of ChatGPT are highlighted across diverse medical domains, including pandemic management, surgical consultations, dental practices, medical education, and disease diagnosis. The study categorizes its findings into eight areas of application, ranging from treatment and patient care to imaging and administrative tools. Central to the focus is balancing AI’s capabilities with the need for human oversight.

Methodology: The research employs a systematic literature review, guided by the PRISMA approach, to analyze the applications and limitations of ChatGPT in healthcare. A total of 82 studies are classified into eight thematic groups, covering treatment, imaging, patient interactions, medical devices, and professional tools. This comprehensive approach allows for an in-depth understanding of the diverse ways ChatGPT can be applied while identifying key challenges and motivations for its use in clinical and educational contexts.

Results: ChatGPT demonstrates significant versatility in healthcare, with applications spanning patient care, diagnostics, education, and administrative support. Its ability to rapidly disseminate information makes it particularly valuable in pandemic management and other time-critical scenarios. However, the study also notes persistent challenges, including the need for human oversight to ensure safety and accuracy. While ChatGPT shows promise as a resource for students, academics, and researchers, its integration into clinical workflows remains limited by technical and ethical concerns.

Practical and ethical implications: The findings highlight ChatGPT’s potential to reshape healthcare by improving efficiency, supporting decision-making, and enhancing patient–professional interactions. It serves as a valuable tool for medical education and research, helping students and academics navigate complex topics. However, ethical considerations remain crucial, particularly regarding the reliability of AI-generated information and the need to preserve human judgment in medical decision-making. Addressing these concerns is essential to ensure the responsible integration of AI tools into healthcare systems while maximizing their innovative potential.

Sacoransky et al. (2023) [33]. ChatGPT and assistive AI in structured radiology reporting: A systematic review.

Focus: The study examines the emerging role of large language models (LLMs), particularly ChatGPT, in structured radiology reporting. Traditionally, artificial intelligence in radiology has centered on image analysis; this research shifts attention to how ChatGPT can contribute to generating structured reports, extracting data from free text, and summarizing radiology findings. The focus lies in exploring ChatGPT’s capacity to enhance accuracy and standardization in radiology while addressing challenges such as data privacy and reliability.

Methodology: A systematic review was conducted using MEDLINE and Embase databases, encompassing studies up to May 2024. Primary studies discussing ChatGPT’s applications in structured radiology reporting were selected based on their relevance. Of 268 articles screened, 8 met the inclusion criteria, providing insights into the model’s capabilities in processing radiology reports and imaging data. This method ensures a targeted evaluation of ChatGPT’s role in a specific domain of healthcare.

Results: The review identified several promising applications for ChatGPT, including transforming unstructured reports into structured ones, extracting relevant data from free text, and generating impressions from imaging findings. All selected studies expressed optimism regarding ChatGPT’s potential to assist radiologists by improving efficiency and standardization. However, recurring concerns were highlighted, such as risks to data privacy, the potential for medical errors, and limitations stemming from ChatGPT’s lack of specialized medical training. These issues underscore the need for further refinement and targeted adaptation of the technology.

Practical and ethical implications: The findings suggest that ChatGPT could significantly transform radiology workflows by improving the consistency and accuracy of reporting while optimizing resource utilization. Integrating advanced features like dynamic few-shot prompting and Retrieval Augmented Generation (RAG) could enhance its utility in diagnostic settings. However, practical implementation must address critical ethical challenges, including safeguarding patient data and ensuring the reliability of AI-generated reports. Transparent research, rigorous validation, and ongoing ethical oversight are essential to align ChatGPT’s capabilities with clinical standards and maximize its potential impact on radiology practice.

Keshavarz et al. (2024) [34]. ChatGPT in radiology: A systematic review of performance, pitfalls, and future perspectives.

Focus: The study systematically reviews the reported performance of ChatGPT in clinical radiology applications, aiming to identify its strengths, limitations, and potential for future integration. Key areas of exploration include ChatGPT’s accuracy in supporting diagnoses, aiding clinical decision-making, and enhancing patient communication, alongside ethical considerations and technical challenges associated with its use.

Methodology: A comprehensive literature review was conducted across four major databases—PubMed, Web of Science, Embase, and Google Scholar—to identify studies using ChatGPT for radiology applications up to January 2024. From 861 studies initially identified, 44 were included in the analysis, evaluating ChatGPT’s performance through various metrics such as accuracy, agreement with radiologists, and responsiveness to complex queries.

Results: The study reveals that 84.1% of the included studies reported high performance by ChatGPT, with notable strengths in diagnostic support and clinical decision-making. A median accuracy of 70.5% was observed in the majority of studies, and agreement with reference standards reached a median of 83.6%. ChatGPTv4 demonstrated significant improvements over v3.5, particularly in handling complex questions and radiology-specific terminology. However, several risks were identified, including biased responses, misinformation, hallucinations, and concerns over data privacy and cybersecurity vulnerabilities. These limitations emphasize the need for further refinement before widespread clinical adoption.

Practical and ethical implications: ChatGPT shows promise in enhancing radiology practices, particularly by supporting diagnostic accuracy and improving communication. However, its limitations—such as the risk of generating inaccurate or biased information—highlight the importance of human oversight. Ethical concerns, including patient privacy and the potential misuse of AI-generated content, must be addressed through stringent regulatory frameworks and robust pre-training protocols. Future research should focus on multicenter studies with diverse datasets to validate ChatGPT’s reliability and guide its integration into radiology workflows responsibly.

Temperley et al. (2024) [35]. Current applications and future potential of ChatGPT in radiology: A systematic review. J Med Imaging Radiat Oncol. 2024;68(3):257–264. doi:10.1111/1754-9485.13621

Focus: This study focuses on evaluating the current utilization and future potential of ChatGPT in radiology, with a primary emphasis on its contributions to decision-making, workflow efficiency, and interdisciplinary collaboration and teaching within healthcare. The research also examines the limitations and challenges of implementing ChatGPT in radiology practice, aiming to provide a comprehensive assessment of its role in the field.

Methodology: The authors conducted a systematic search across PubMed, EMBASE, and Web of Science databases to identify relevant studies. The review included six prospective studies, which assessed 551 ChatGPT evaluation events spanning versions 3.0 to 4.0. These studies focused on key areas such as ChatGPT’s ability to handle complex decision-making, improve workflow, and facilitate collaboration, as well as its performance in answering radiology-related questions.

Results: The results revealed several strengths and limitations of ChatGPT. It demonstrated an ability to enhance decision-making and optimize workflow, especially when responding to lower-order thinking questions, with a significant improvement in accuracy from version 3.5 to version 4.0, particularly in imaging-related queries (accuracy increased from 61% to 85%, *p* = 0.009). However, ChatGPT’s performance in generating accurate academic papers and responding to questions about interventional radiology procedures was suboptimal, with data inaccuracies occurring 80% of the time and 45% of responses containing entirely incorrect information. Additionally, ChatGPT’s performance in interpreting CT and MRI findings received a high translation ability score of 4.27/5 on the Likert scale.

Practical and ethical implications: The study highlights ChatGPT’s significant potential to augment decision-making processes and optimize radiology workflows. However, its current limitations—such as frequent inaccuracies in generating academic content and providing detailed radiology procedure information—underscore the need for rigorous validation and continuous evaluation. Ethical considerations, including the reliability of AI-generated information and the necessity for clinical oversight, are critical before adopting ChatGPT widely in clinical settings. These findings suggest that while ChatGPT offers promising benefits, it should be used cautiously and undergo thorough validation in diverse clinical environments to ensure its safe and effective integration into radiology practice.

Gorenstein et al. (2024) [36]. Bidirectional Encoder Representations from Transformers in Radiology: A Systematic Review of Natural Language Processing Applications.

Focus: This study evaluates the influence and applications of Bidirectional Encoder Representations from Transformers (BERTs) within the field of radiology. Since its introduction in 2018, BERTs have revolutionized natural language processing (NLP) with their bidirectional context understanding, enabling innovative uses in the radiologic domain. The study aims to assess how BERT-based models are applied to various radiology tasks, particularly in report classification and information extraction.

Methodology: The authors conducted a systematic review following the Preferred Reporting Items for Systematic Reviews and Meta-Analyses (PRISMA) guidelines. The search focused on the literature published from 1 January 2018 to 12 February 2023, and specifically targeted studies on BERT-based models and NLP in radiology. The search strategy included terms related to generative models, transformer architecture, and different imaging techniques. A total of 30 studies met the inclusion criteria after screening 597 results.

Results: Among the included studies, the majority were retrospective, with 14 of them published in 2022. The studies primarily focused on the use of BERTs in classification and information extraction from radiology reports, with chest X-rays being the most commonly studied imaging modality. Specific applications investigated included automatic CT protocol assignment and the use of deep learning models for interpreting chest X-rays. These applications demonstrated the utility of BERTs in improving the efficiency of report generation and enhancing the accuracy of diagnostic processes.

Practical and ethical implications: The study emphasizes the practical applications of BERTs in radiology, particularly in streamlining tasks like report classification, protocol assignment, and even report generation. As BERT technology continues to evolve, it holds significant promise for enhancing diagnostic precision, reducing the time required for report generation, and ultimately improving patient care. However, the use of AI-based technologies like BERTs also raises ethical considerations, particularly around data privacy, the need for transparency in AI-driven decision-making, and ensuring that AI tools are used to support, not replace, clinical judgment. Ongoing evaluation of these technologies will be crucial for their safe and effective integration into clinical practice.

Diab et al. (2023) [37]. Natural Language Processing for Breast Imaging: A Systematic Review.

Focus: The study investigates the role of natural language processing (NLP) in diagnostic radiology, particularly its applications in breast imaging. With the increasing prominence of NLP, the study explores how these technologies can enhance various aspects of breast cancer care, including triage, diagnosis, lesion characterization, and treatment management. The review aims to provide a comprehensive overview of the techniques used to extract valuable information from clinical notes, radiology reports, and pathology reports, emphasizing their potential to improve both the accuracy and efficiency of breast imaging.

Methodology: The review systematically examines recent advancements in NLP as applied to breast imaging. It evaluates various NLP methods used to extract information from key medical documents such as clinical notes, radiology reports, and pathology reports. The study further explores the state of the art in NLP-based decision support systems for breast imaging, identifying their benefits and the challenges that remain. Although the specific methodology used for selecting studies is not detailed, the review offers an in-depth analysis of the current literature in the field.

Results: The review highlights significant advancements in the use of NLP for breast imaging, showing its potential to improve diagnostic accuracy and streamline processes. The use of NLP in extracting relevant information from clinical notes and reports helps enhance the efficiency of diagnosing and characterizing lesions. Moreover, NLP-based decision support systems for breast imaging have emerged as promising tools, although there are still several challenges, such as integrating these technologies into clinical practice and ensuring their effectiveness. The study points to the future potential of NLP in improving patient care, with increased accuracy in diagnoses and a more efficient workflow.

Practical and ethical implications: The integration of NLP into breast imaging practices offers substantial practical benefits, including improved diagnostic accuracy and more efficient triage and treatment management. By automating the extraction of critical information from medical reports, NLP can reduce clinician workload and speed up decision-making, ultimately enhancing patient care. However, the adoption of these technologies raises ethical issues, particularly concerning the accuracy of NLP algorithms, potential bias in data processing, and the risk of over-reliance on AI-driven systems. Ensuring transparency, mitigating bias, and maintaining clinician oversight are crucial to the safe implementation of NLP in breast imaging care.

Saha A et al. (2023) [38]. A scoping review of natural language processing of radiology reports in breast cancer.

Focus: The study examines the application of natural language processing (NLP) algorithms in analyzing radiology reports related to breast cancer diagnosis and care. It highlights various uses of NLP, including cohort selection for clinical trials, populating large-scale data registries, and improving radiology workflows, particularly in mammography screening. This study classified as systematic review article is the first to specifically focus on the applications of NLP within the context of breast cancer, providing a comprehensive overview of the field.

Methodology: A total of 210 articles were initially identified, of which 44 met the inclusion criteria for this review. The selected studies involved the development or evaluation of NLP algorithms applied to free-text radiology reports of breast cancer. The data extracted from these studies included both clinical and technical aspects of the algorithms, providing insights into their effectiveness and applications.

Results: The review found that the majority of studies focused on using NLP for diagnostic and screening processes, with fewer studies addressing treatment or therapeutic applications. Over the years, there has been an increase in the use of advanced techniques such as deep learning and transfer learning, although rule-based approaches remain useful in certain contexts. Additionally, there has been a noticeable increase in efforts to share code and software, although data sharing remains limited, which could impede broader collaboration and the validation of NLP models.

Practical and ethical implications: The practical benefits of NLP in breast cancer care are significant, particularly in improving diagnostic accuracy and enhancing screening processes, such as mammography interpretation. The integration of NLP can streamline workflows, improve the efficiency of clinical trial selection, and help populate large data registries. However, the ethical implications are multifaceted. Challenges related to data privacy, especially when sharing large datasets, and the need for transparency in the development of algorithms are key concerns. Furthermore, the reliance on NLP models should be carefully balanced with human expertise to ensure clinical decisions remain patient-centered and accurate.

Pons et al. (2016) [39]. Natural Language Processing in Radiology: A Systematic Review. Radiology.

Focus: This study explores the use of natural language processing (NLP) techniques to extract structured data from the large volumes of free-text radiology reports stored in electronic health records. The study highlights the potential of these reports as a valuable source of information for enhancing clinical care and supporting research. However, the challenge lies in efficiently converting unstructured, free-text data into structured formats that can be effectively utilized by computers.

Methodology: The study conducted a systematic literature search, identifying 67 relevant publications that describe various NLP methods applied to radiology. These methods aim to automatically identify and extract key information from radiology reports, transforming the free-text narrative into structured data that can be used for various purposes.

Results: The review highlights various applications of NLP in radiology, focusing on the tasks being performed (such as information extraction), the NLP methodologies and tools employed, and the results of their application. The study also discusses the limitations of current NLP methods and the challenges in advancing these techniques for broader use in radiology. These include issues related to accuracy, adaptability to different radiology report formats, and the need for further research and development to improve performance.

Practical and ethical implications: The potential for NLP to enhance clinical care by enabling the automatic extraction of meaningful data from radiology reports is significant. This could improve decision-making, support research, and enhance workflow efficiency. However, the challenges associated with accuracy, the complexity of medical language, and the need for standardized reporting practices are key obstacles. Ethical considerations around patient data privacy and the transparency of NLP algorithms also remain crucial as the technology develops.

Linna and Kahn (2022) [40]. Applications of natural language processing in radiology: A systematic review.

Focus: The study systematically reviewed the trends and applications of natural language processing (NLP) in radiology over the past five years, particularly focusing on the use of NLP techniques, including machine learning, and their effectiveness in various radiology applications.

Methodology: The study conducted a search of three peer-reviewed databases—PubMed, EMBASE, and Web of Science—covering publications from 1 January 2016 to 21 April 2021. A total of 228 studies were included in the review. The studies were analyzed based on factors such as clinical application, study setting, NLP techniques used, and performance metrics.

Results: The review found an increase in studies involving NLP in radiology, with a significant rise in the use of machine learning models such as deep learning and transformers. More than 50% of the studies reported an F1 score greater than 0.91, demonstrating high performance. The sample sizes varied across studies, with a median of 3708 data points, mostly derived from radiology reports. NLP applications were categorized as clinical (87 studies), technical (66 studies), quality improvement (61 studies), research (9 studies), and education (5 studies). A majority (145 studies) utilized data from a single academic center.

Practical and ethical implications: The review highlights the significant potential of advanced NLP techniques, such as deep learning- and transformer-based models, to improve radiology tasks, both interpretative and non-interpretative. Practically, further work is needed to enhance the clinical applicability and portability of NLP systems across various healthcare settings to bridge the gap between research and real-world clinical use, ultimately improving patient care and workflow efficiency. Ethically, there are concerns regarding data privacy, consent, and algorithmic bias. Ensuring fairness, transparency, and the responsible use of NLP in clinical environments is crucial to mitigate risks and promote equitable outcomes.

Davidson et al. (2021) [41]. The reporting quality of natural language processing studies: systematic review of studies of radiology reports.

Focus: This systematic review aimed to summarize the characteristics and reporting quality of studies applying natural language processing (NLP) to radiology reports. The goal was to evaluate how NLP is used in extracting health information from radiology reports and to assess the transparency and reproducibility of these studies.

Methodology: The study searched Google Scholar for studies published in English between January 2015 and October 2019 that applied NLP to radiology reports of any imaging modality. At least two reviewers independently screened the studies and conducted data extraction. The review focused on 15 criteria related to data sources, datasets, ground truth, outcomes, and reproducibility for quality assessment. Primary NLP performance measures included precision, recall, and F1 score.

Results: Out of 4836 records retrieved, 164 studies were included in the review. The most common clinical applications of NLP were disease classification (28%) and diagnostic surveillance (27.4%). The majority of studies (86%) used English radiology reports, and 28% used reports from mixed imaging modalities. Oncology was the most frequently studied disease area (24%). Most studies had a dataset size greater than 200 (85.4%), but only a portion described the annotated, training, validation, and test sets (67.1%, 63.4%, 45.7%, and 67.7%, respectively). Performance metrics such as precision (48.8%) and recall (53.7%) were reported in about half of the studies. However, few studies reported external validation (10.8%), data availability (8.5%), or code availability (9.1%). No clear correlation between reporting quality and NLP performance was identified.

Practical and ethical implications: From a practical standpoint, the review highlights that NLP has significant potential for clinical applications in radiology, especially in disease classification and diagnostic surveillance. However, the lack of standardized reporting and transparency in the studies limits the ability to compare results and replicate findings, which poses a barrier to broader clinical implementation. To ensure the effective use of NLP in radiology, it is crucial to develop and adopt reporting standards that improve the quality, transparency, and reproducibility of studies. Ethically, the variability in data reporting, the lack of external validation, and limited access to code raise concerns about the reproducibility and accountability of NLP models. These issues need to be addressed to ensure that NLP technologies are reliable, transparent, and can be used ethically in clinical practice without compromising patient care.

Casey et al. (2021) [42]. A systematic review of natural language processing applied to radiology reports.

Focus: This study systematically reviews and quantifies recent literature on the application of natural language processing (NLP) to radiology reports. The aim is to assess the advancements in using NLP to extract structured information from radiology reports and to provide a comprehensive analysis of recent developments in this area.

Methodology: An automated literature search was conducted, yielding 4836 results. The search was enhanced with metadata enrichment, citation tracking, and manual review. A total of 164 publications were included in the analysis. The study evaluated these publications based on 21 variables, including radiology characteristics, NLP methodology, performance metrics, and clinical application characteristics.

Results: The study found a significant increase in publications, with the number of papers in 2019 nearly tripling those in 2015. The included studies were categorized into six clinical application categories. While deep learning techniques have seen an increase in use, conventional machine learning approaches remain dominant. Deep learning methods continue to face challenges, particularly when data are scarce, and there is limited evidence of these methods being adopted into clinical practice. Despite 17% of studies reporting F1 scores greater than 0.85, the ability to make comparative evaluations is difficult due to the use of different datasets across studies. Data sharing was limited, with only 14 studies making their data available, 15 providing code, and 10 validating results externally.

Practical and ethical implications: This review underscores NLP’s potential to improve healthcare workflows by automating the interpretation of radiology reports. However, challenges such as reproducibility, explainability, and clinical adoption remain. Enhancing the sharing of data and code is essential for external validation, reducing variability, and improving NLP model robustness, which will support broader implementation in clinical practice. The limited sharing of data and code raises ethical concerns about transparency and the reliability of NLP models. Without open access, ensuring the ethical integrity and trustworthiness of these models becomes difficult, potentially compromising patient care. The lack of external validation further increases the risk of unverified models being used in clinical practice, highlighting the need for responsible and ethical deployment of NLP technologies in healthcare.

Sorin et al. (2020) [43]. Deep Learning for Natural Language Processing in Radiology-Fundamentals and a Systematic Review.

Focus: This review explores the application of deep learning-based natural language processing (NLP) in radiology, particularly its ability to convert free text into structured data. The study aims to survey the fundamentals of deep learning NLP and assess its use in radiology, highlighting recent innovations that enhance NLP performance.

Methodology: A systematic review was conducted following the Preferred Reporting Items for Systematic Reviews and Meta-Analyses (PRISMA) guidelines. The study searched for deep learning NLP radiology studies published up to September 2019 across MEDLINE, Scopus, and Google Scholar databases. A total of ten relevant studies published between 2018 and 2019 were identified for analysis.

Results: The review identified several deep learning models used in NLP for radiology, including convolutional neural networks (CNNs), recurrent neural networks (RNNs), long short-term memory networks (LSTMs), and attention networks. These models were applied to tasks such as diagnosing conditions like pulmonary embolisms and fractures, labeling follow-up recommendations, and selecting imaging protocols. Deep learning models performed as well as or outperformed traditional NLP models, indicating significant improvements in NLP applications for radiology.

Practical and ethical implications: The review underscores the growing use and potential of deep learning NLP in radiology to streamline tasks like diagnostic flagging and imaging protocol selection. As these technologies evolve, it is essential for radiologists to become familiar with deep learning NLP tools, as they are expected to play a significant role in enhancing clinical workflows and improving diagnostic accuracy in the near future. With the increasing use of deep learning NLP in radiology, ethical concerns arise regarding the transparency and accountability of these models. While the technology improves performance, ensuring that these systems are ethically reliable and explainable is crucial to avoid potential risks to patient care. There is a need for ethical oversight to address concerns about model bias, decision-making transparency, and the potential for errors affecting patient outcomes. Ensuring the responsible use of these technologies is essential for maintaining trust in their clinical applications.

Blackley et al. (2019) [44]. Speech recognition for clinical documentation from 1990 to 2018: a systematic review.

Focus: This study aimed to review the recent literature on the use of speech recognition (SR) technology for clinical documentation also with the focus in this field. It sought to assess the impact of SR on factors such as document accuracy, provider efficiency, and institutional costs, focusing particularly on its application in various medical departments.

Methodology: The study conducted a systematic review of articles published between January 1990 and October 2018. It searched 10 scientific and medical literature databases for articles that investigated the use of SR by clinicians for documentation purposes. The selected articles were analyzed based on their research topics, medical domains, and the specific SR systems evaluated.

Results: A total of 122 articles were included in the review. Of these, 48 (39.3%) focused exclusively on radiology, while 10 (8.2%) involved emergency medicine. A significant portion of the studies (39.3%) evaluated productivity, with 16.4% focusing on the impact of SR on documentation time, which showed mixed results. However, all studies that assessed turnaround time (15.6%) reported improvements. Error analyses were conducted in 23.8% of the studies, with error rates ranging from 4.8% to 71%. Some studies (5.7%) evaluated costs, with mixed findings—with five reporting cost reductions and two reporting increases. Nuance Communications was the most frequently used SR product (44.3%), followed by IBM (9.0%) and Philips (6.6%).

Practical and ethical implications: The findings highlight the widespread use of SR technology in clinical documentation, particularly in radiology, but also extending to other departments like emergency medicine. Despite its adoption, the research on SR remains heterogeneous, with varied evaluation metrics and inconsistent results. While SR has demonstrated potential to improve turnaround times and productivity, the error rates in documentation are concerning, ranging significantly across studies. Furthermore, the impact of SR on institutional costs is still not fully understood, as findings are mixed. From an ethical perspective, the variability in error rates and the lack of standardized metrics for evaluating SR effectiveness raise concerns about the reliability and accountability of SR-assisted documentation. To enhance the impact of SR technology, there is a need for further research to standardize evaluation criteria, ensure high-quality documentation, and assess the broader implications of SR use in clinical settings.

Dias et al. (2019) [45]. Using Machine Learning to Assess Physician Competence: A Systematic Review.

Focus: This study aimed to identify the machine learning (ML) techniques applied to automate physician competence assessments and evaluate their use in assessing different competence domains across several medical specialties. The review sought to understand how these techniques are implemented and how they might improve real-time assessments of physicians’ competencies.

Methodology: The study conducted a systematic search in multiple databases, including MEDLINE, EMBASE, PsycINFO, Web of Science, ACM Digital Library, IEEE Xplore Digital Library, PROSPERO, and the Cochrane Database of Systematic Reviews. Articles published from the inception of these databases until 30 April 2017 were considered. Studies were included if they used at least one ML technique to assess the competence of medical students, residents, fellows, or attending physicians. Information such as sample size, participants, study design, medical specialty, ML techniques, competence domains, and outcomes were extracted. The methodological quality was evaluated using the MERSQI tool, and a qualitative narrative synthesis of the data was performed.

Results: Of the 4953 articles initially reviewed, 69 met the inclusion criteria. The most studied specialties were general surgery (24 studies, 34.8%) and radiology (15 studies, 21.7%). The ML techniques most frequently applied were natural language processing (24 studies, 34.8%), support vector machines (15 studies, 21.7%), and hidden Markov models (14 studies, 20.3%). The competence domains most assessed were patient care (63 studies, 91.3%) and medical knowledge (45 studies, 65.2%).

Practical and ethical implications: The study indicates a growing interest in using ML techniques for physician competence assessment, particularly in general surgery and radiology. The most common techniques, such as natural language processing, support vector machines, and hidden Markov models, show promise in assessing key competence domains like patient care and medical knowledge. However, the study emphasizes the need for further validation research to ensure the reliability and effectiveness of these techniques in clinical settings. From a practical perspective, ML techniques may facilitate real-time assessment and intervention by integrating and analyzing data efficiently. Ethically, the widespread use of ML in physician assessment could enhance objectivity and reduce biases. However, ensuring the transparency, fairness, and accuracy of these models is critical to maintaining the integrity of the assessment process and ensuring equitable outcomes for all physicians.

#### 3.1.3. Addressing Challenges and Providing Clear Recommendations for Future Research in the Application of Natural Language Processing in Radiology

The analysis of the studies presented in the previous section highlights a series of practical challenges facing the application of natural language processing (NLP) in radiology. These challenges, categorized into key areas such as data sharing, model validation, standardization of reporting practices, and ethical considerations, emerge from the detailed examination of the studies reviewed.

*Data Sharing and Privacy Concerns*

A key limitation identified is the difficulty in accessing diverse, high-quality datasets due to privacy restrictions. As emphasized by Klug et al. [31], the development of robust NLP models requires comprehensive datasets from a variety of patient populations, which is often hindered by privacy laws and institutional barriers. To overcome this, federated learning and synthetic data generation are proposed as promising solutions [32,33]. These technologies allow for collaborative model training without the need to transfer sensitive patient data, thus maintaining privacy while providing the data necessary for effective model development.

*Model Validation and External Evaluation*

Validation of NLP models in radiology also presents challenges, with models often performing well in research settings but failing to maintain their accuracy in real-world clinical environments. Variations in data formats, terminology, and reporting styles across institutions make it difficult for NLP systems to generalize effectively. To address this, Sorin et al. [43] advocate for external validation across diverse clinical contexts. Standardized protocols for validating NLP models, including collaborations between academic and clinical partners, could foster the development of benchmark datasets and evaluation metrics that ensure models are adaptable to multiple clinical settings.

*Standardization of Radiology Reporting*

A major issue for NLP in radiology is the lack of standardization in reporting practices. Unstructured reports, in particular, complicate the extraction of useful data for NLP systems. Diab et al. [37] highlight this challenge, stressing the need for standardized reporting formats. Structured reporting could improve the reliability and consistency of radiology reports, making it easier for NLP systems to process them. Pons et al. [39] support this by demonstrating the positive impact of structured reporting on the performance of NLP systems, suggesting that future studies should explore the widespread adoption of standardized formats across institutions.

*Ethical Considerations and Algorithmic Transparency*

Ethical concerns, including the potential for algorithmic biases and the lack of transparency in some NLP models, also arise from the studies analyzed. Gorenstein et al. [36] stress the importance of transparency to ensure trust in NLP technologies and prevent the perpetuation of healthcare disparities. Future research should prioritize explainability in NLP models and implement bias mitigation strategies, such as diversifying training datasets and using fairness algorithms. These steps are essential for ensuring equitable outcomes and maintaining patient trust.

*Concrete Recommendations for Future Research*

Based on the challenges identified, the following concrete recommendations are proposed:

Development of Privacy-Preserving Data-Sharing Methods: Federated learning and synthetic data generation should be further explored as solutions to facilitate collaborative model training while preserving patient privacy. These approaches will enable the creation of more diverse datasets and improve model performance across varied populations [32,33].

Establishment of External Validation Protocols: Research should focus on establishing standardized validation protocols to assess NLP models across different clinical settings. This will ensure that the models are adaptable to a range of report formats and institutional settings, increasing their generalizability and reliability [43].

Standardization of reporting practices: Promoting the adoption of structured reporting formats is critical for improving the consistency of radiology reports and enhancing the ability of NLP systems to process them. The integration of standardized medical terminologies will further improve the accuracy and efficiency of NLP applications [39].

Ethical considerations and bias mitigation: Future studies should focus on improving the transparency and interpretability of NLP models, enabling clinicians to understand and trust the results. Additionally, efforts should be made to reduce biases in NLP systems, ensuring that these technologies do not perpetuate existing healthcare disparities [36].

By addressing these challenges and implementing the proposed solutions, the potential for NLP technologies in radiology can be fully realized, leading to improved clinical workflows and patient outcomes.

### 3.2. Trends

An analysis of trends in a biomedical database offers valuable insights into the integration of ethical considerations in algorithmic research, particularly within the healthcare sector. For this analysis, we selected PubMed, which serves as a representative example of broader trends in this field.

Using the composite search key outlined in Box 1, position 1, we identified 713 studies dating back to 1993. The early date (1993) is noteworthy, indicating that chatbot systems such as Automated Response Systems (ARSs), Virtual Assistants (VAs), and Automated Bots (ABSs) were being explored and implemented in healthcare well before the widespread adoption of artificial intelligence (AI) technologies.

**Figure 2:**
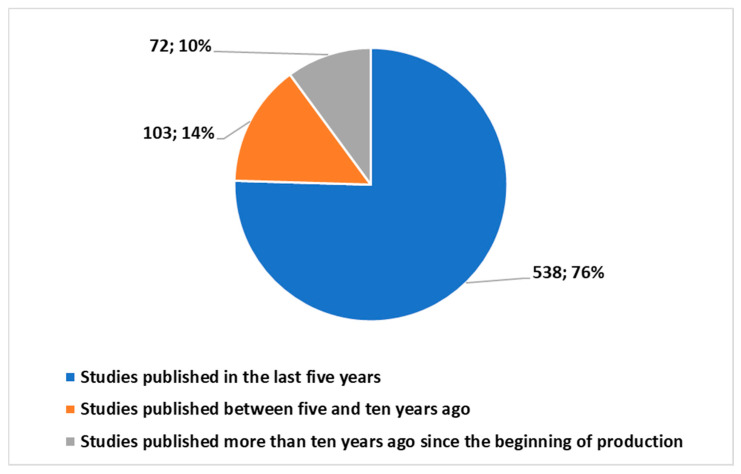
Growth of publications from 1993 to present.

A temporal analysis reveals a significant surge in research in the past five years, driven largely by the COVID-19 pandemic (Figure 2). The pandemic accelerated the need for remote and automated healthcare solutions, which likely contributed to the sharp increase in studies. Over the last decade, a total of 641 studies were produced, accounting for 90% of the total number of studies in this field. Narrowing the timeframe to the past five years, the number of studies rises to 538, comprising 75.5% of the total.

**Figure 3:**
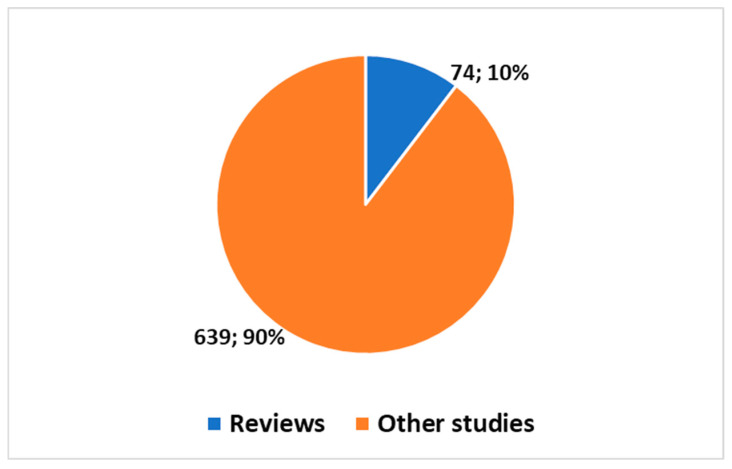
Distribution of review articles (1993-present).

A more detailed breakdown shows that 74 studies, or 10.4%, were dedicated to reviews and systematic reviews (Figure 3). Among these, 15 studies were systematic reviews produced after 2016, reflecting a growing emphasis on evaluating and consolidating the existing body of research, and highlighting the increasing importance of critically assessing the use of chatbot systems and AI in healthcare. The surge in research during the past five years, especially in light of the COVID-19 pandemic, underscores the urgent need for scalable and efficient healthcare solutions. The rapid deployment of telehealth, remote diagnostics, and automated response systems during the pandemic has fundamentally reshaped the way healthcare is delivered. As AI and chatbot systems become more prevalent, ethical concerns—such as patient data privacy, algorithmic bias, and the transparency of decision-making processes—are becoming increasingly critical. This makes the growing body of systematic reviews particularly important as they help consolidate knowledge and guide future innovations in ethically responsible ways.

Additionally, the historical context of chatbot usage in healthcare, dating back to 1993, provides an interesting perspective on how foundational these technologies are, even though their mainstream recognition has occurred more recently. This early exploration demonstrates that the healthcare sector has long been at the forefront of digital innovation, albeit with varying levels of integration and acceptance over the years.

Box 1. **The proposed composite search keyword.**

*((chatbot[Title/Abstract]) OR (NLP[Title/Abstract]) OR (chatgpt[Title/Abstract]) OR(natural language processing[Title/Abstract]) OR (natural language model[Title/Abstract])) AND (radiology[Title/Abstract])*

### 3.3. Outcome of This Umbrella Review with a Focus on Specific Aims

This section explores the intersection of NLP and chatbots in radiology answering the specific aims of the overview. It includes an evaluation of their impact and identifies key opportunities and challenges. The overview aims to offer valuable insights into optimizing the design and governance of AI systems in radiology, specifically focusing on the use of natural language processing and chatbot applications.

#### 3.3.1. Evaluate Contributions in the Field of NLP/NLM/Chatbots in Radiology

##### Common Message: The Impact of Chatbots and NLP in Radiology

The integration of NLP technologies into radiology is revolutionizing medical diagnostics. NLP-driven tools are transforming the analysis and processing of digital diagnostic data, leading to significant improvements in accuracy, efficiency, and accessibility in healthcare. This discussion will discuss how NLP interacts in radiology, improving workflows and supporting public health initiatives, highlighting its critical role in advancing radiology practices.

*Improving the patient journey*: The application of NLP has the potential to enhance clinical decision-making systems and improve the overall patient journey. The study by Klug et al. [31] emphasizes that radiology reports are among the most extensively studied document types in clinical NLP research. However, many other stages of the patient journey, such as treatment and aftercare, remain relatively unexplored. Expanding NLP research beyond radiology could lead to significant advancements in clinical decision-making and patient outcomes.

*Application of ChatGPT in radiology*: ChatGPT and assistive AI have substantial potential to transform radiological reporting by improving accuracy, standardization, and resource optimization. The review by Younis et al. [32] reports that radiology is one of the major areas where ChatGPT is having a significant impact on healthcare. The study by Sacoransky et al. [33] highlights that those recent advancements, especially with transformer-based models like ChatGPT, are promising for improving structured radiology reporting. A review of eight studies demonstrated ChatGPT’s ability to generate structured reports and extract data, though issues with data privacy and reliability remain. Future advancements may involve integrating ChatGPT with dynamic prompting and Retrieval Augmented Generation (RAG) to further enhance diagnostic workflows. Additionally, research by Keshavarz et al. [34] underscores ChatGPT’s effectiveness across various radiology applications but calls for more comprehensive studies to address its limitations and confirm its accuracy. Notably, ChatGPT-4 has demonstrated significant improvements over ChatGPT-3.5 in handling complex queries, understanding radiology terminology, and accurately describing images. These enhancements were highlighted in the systematic review by Temperley et al. [35], which noted that while ChatGPT shows promise in improving decision-making, workflow efficiency, and interdisciplinary collaboration in radiology, it also has limitations, including high rates of data inaccuracies and incorrect information on interventional procedures.

*Impact of BERTs on radiology*: Introduced in 2018, BERTs (Bidirectional Encoder Representations from Transformers) have enabled innovative applications. BERTs have significantly advanced NLP in radiology and enable bidirectional understanding of word context, especially in report classification and information extraction. The study by Gorenstein et al. [36] highlights the use of BERTs in the automation of Computed Tomography (CT) protocol assignments and the interpretation of chest radiographs. Further development of BERT technology promises further improvements in diagnostic accuracy, reporting, and the optimization of patient care.

*Application of NLP in breast imaging and cancer care*: Breast imaging encompasses a variety of medical imaging procedures aimed at examining breast tissue for the early detection and diagnosis of breast disease, particularly breast cancer. The review by Diab et al. [37] highlights the growing role of NLP in breast imaging and its potential to improve triage, diagnosis, lesion characterization, and treatment management. The review describes various NLP techniques for extracting key information from clinical notes and radiological and pathological reports and demonstrates their positive impact on the accuracy and efficiency of breast cancer diagnosis. It also explores the challenges and future opportunities for more effective integration of NLP into breast imaging workflows. Similarly, Saha et al. [38] investigated NLP applications in breast cancer care, focusing on its use in diagnostic and screening workflows, with emerging deep learning (DL) approaches improving the efficiency and accuracy of radiology reports. Both reports emphasize the potential of NLP to streamline breast cancer diagnostics but also identify challenges such as limited data sharing.

*Multiple uses of NLP in radiology*: NLP has a wide range of applications in clinical radiology, and several reports have addressed this topic and emphasized its versatility in this field. The study by Pons et al. [39] looks at the use of NLP in radiology and emphasizes its role in converting free-text radiology reports into structured data. The study identifies five key areas where NLP is used: diagnostic surveillance, cohorting for epidemiological studies, query-based case retrieval, quality assessment of radiological practice, and clinical support services. In contrast, the study by Linna et al. [40] focuses on the trends in NLP applications in radiology over the last five years and emphasizes the increasing use of advanced techniques such as deep learning and transformers. Although NLP models demonstrate high performance, the report emphasizes the need for broader clinical implementation and transferability to fully exploit their potential to improve patient care. These applications have been categorized into clinical, technical, quality improvement, research, and educational areas.

*NLP applied to radiology reports*: The application of NLP to radiology reports has been analyzed in detail in several systematic reviews. The study by Davidson et al. [41] focused on the quality and transparency of reporting in NLP research. It concluded that although NLP has important clinical applications, such as disease classification and diagnostic monitoring, the overall quality of reporting is suboptimal and hinders reproducibility and comparability. The study argues in favor of the development of specific reporting standards for clinical NLP research. Of note, 28% of the studies analyzed used reports from mixed imaging modalities, with oncology being the most common focus (24%). This review emphasizes the broad potential of NLP in radiology for both clinical services and research. The review by Casey et al. [42] also shows that despite the high performance of NLP models, but there are still challenges. Problems such as data scarcity, reproducibility, and limited clinical acceptability remain significant obstacles. The study calls for increased efforts in code sharing and the standardization of methods to improve comparability and facilitate the integration of NLP techniques into radiology.

*Advancements in deep learning for NLP in radiology*: Advances in DL have been instrumental in improving NLP applications in radiology, as described in the review by Sorin et al. [43]. This study highlights the advances in DL techniques that have surpassed traditional methods. It examines ten studies published between 2018 and 2019, focusing on important applications such as identifying dual diagnoses such as pulmonary embolism and fractures, labeling recommendations for follow-up, and automating the selection of imaging protocols. The report emphasizes the growing effectiveness and acceptance of DL models, which significantly improve NLP performance in radiology and promote more accurate, automated clinical workflows.

*Use of speech recognition*: The use of speech recognition (SR) technology for clinical documentation has grown significantly in recent years. The review by Blackley et al. [44] provides a comprehensive overview of SR applications in clinical settings, revealing that while SR is widely adopted, the research on its effectiveness is fragmented, with inconsistent evaluation methods and mixed findings. The study also highlights the expanding use of SR beyond radiology into other medical domains, underscoring the need for further investigation into its efficiency, accuracy, and overall impact on clinical workflows and patient care.

*Assessment of physician competence*: Artificial intelligence (AI) is increasingly being used to train and assess the core competences of physicians. The study by Dias et al. [45] investigated different machine learning techniques used to assess the competence of physicians in different medical specialties. General surgery and radiology were the most commonly studied areas, with NLP and support vector machines. While many studies demonstrate the feasibility of these approaches, the study highlights the need for further validation to improve real-time assessments and interventions and ultimately aims to integrate AI into everyday clinical practice to make competency assessment more effective.

Table 1 provides a sketch of key studies that examine the role of chatbots and natural language processing (NLP) in radiology. The table offers an outline of the primary aims and focus areas of these studies, shedding light on how AI technologies, such as chatbots and NLP, are being integrated into radiological practices. This categorization touches on themes like clinical decision-making, workflow optimization, structured reporting, and diagnostic support, offering a preliminary look into the evolving role of these technologies in the field.

#### 3.3.2. AI Applications in Radiology: Opportunities and Areas Needing a Broader Investigation

An interesting perspective that emerges from the studies is not only the detailed focus and categorization of each individual study, but also the identification of broader thematic areas related to the integration of AI and the intersection of natural language processing (NLP) in radiology. This dual approach allows us to recognize both the specific contributions of individual studies and the overarching trends spanning multiple research papers. By examining both micro-level details and macro-level themes, we gain a comprehensive understanding of how AI and NLP research in radiology influence and complement each other, creating a more nuanced and interconnected landscape.

This analysis not only highlights the opportunities provided by integrating AI and NLP into radiological practice but also identifies areas that require improvements. Areas in need of attention emerge clearly from the detailed categorization, providing insights into how challenges can be addressed and the effectiveness of these technologies enhanced.

Table 2 categorizes broad areas related to the application of technologies such as ChatGPT and natural language processing (NLP) in radiology, outlining their respective opportunities and limitations. This table aims to provide a concise overview of how these innovations can enhance radiological practices while also highlighting the challenges that must be addressed for effective implementation. By summarizing key references, the table offers insights into the potential impact of these technologies on clinical workflows, reporting quality, competence assessment, and overall improvements in diagnostics and patient care, as well as facilitating a structured understanding of future research and development needs.

### 3.4. The Synoptic Diagram

**Figure 4:**
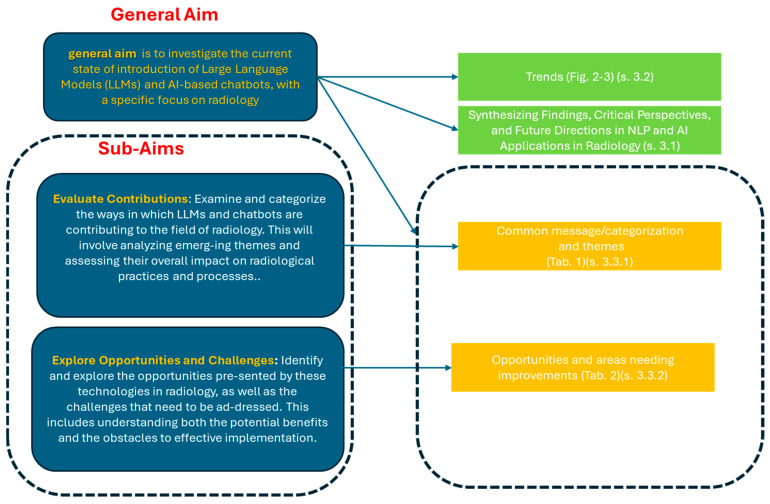
Synoptic diagram presenting an editorial overview of the key tabular/graphical highlights from the results.

Figure 4 provides a concise schematic overview of the outcome of this umbrella review, organized into tabular connections and diagrams that align with the overall progression of the discourse. Based on the general and specific aims proposed in this review, Block 1 (top right) outlines the emerging trends as reported graphically in Figure 2 and Figure 3 (Figure 2 and Figure 3 in the synoptic). Block 2 (below) highlights the common message/opportunities and the categorization as elucidated in Table 1 (Table 1 in the synoptic), while Block 3 (continuing below) elucidates by means of the Table 2 (Table 2 in the synoptic) the opportunities and areas needing improvements. Related ections (indicated with s.X.X. are also reported in the synoptis)

## 4. Discussion

The discussion is organized into six comprehensive subsections, each addressing a critical aspect of this study’s findings and implications.

Section 4.1 aligns this study with its initial objectives, critically evaluating how well the outcomes match the predefined goals. This section emphasizes the added value the research brings to the existing body of knowledge, showcasing how this study enhances understanding and addresses the research questions established at the outset. By providing clear comparisons between the expected and actual outcomes, this subsection highlights the significance of the findings in advancing the field.

Section 4.2 presents the direct and indirect recommendations emerging from the analyzed studies. These recommendations are categorized to reflect their nature and intended impact. Direct recommendations focus on specific actions that can be taken to improve the implementation of NLP/NLM technologies in clinical settings. In contrast, indirect recommendations address broader systemic issues that must be considered to facilitate effective integration and long-term success. By clearly delineating these two categories, this subsection underscores the importance of both immediate actions and broader contextual considerations in shaping future research and practice.

Section 4.3 extends the discussion by incorporating insights from recent cross-sectional studies across various domains beyond radiology. This examination includes perspectives on ethics, collaboration, and regulation, drawing connections between these fields and the application of NLP/NLM technologies. By exploring how ethical considerations and collaborative practices can enhance the effectiveness of these technologies, this subsection enriches the discussion and underscores the multifaceted nature of the challenges and opportunities in this area.

Section 4.4 provides a synoptic overview that presents an editorial summary of the key tabular highlights from this study. This section organizes and synthesizes the main findings into a clear and accessible format, facilitating a comprehensive understanding of this study’s critical data points and conclusions. By summarizing the key results visually, this subsection ensures that readers can quickly grasp the essential insights and implications of the research.

Section 4.5 provides a final reflection emphasizing that ethics is a critical priority for adopting NLP and chatbot technologies in radiology, aligning with the emerging field of “algorethics”. It highlights the role of international frameworks, such as those from WHO, the EU, and FDA, in guiding ethical standards, ensuring that these technologies are implemented responsibly and transparently to protect patient rights and foster equity.

Finally, Section 4.6 outlines the limitations of this study.

### 4.1. Discussion on the Added Value of This Umbrella Review and Alignment with the Purpose

This umbrella review systematically organizes findings into key subsections, providing a comprehensive understanding of the landscape surrounding NLP and chatbots in radiology. This structured approach enhances the visibility of prevailing trends and underscores the significance of these technologies over time. For instance, Section 3.1 highlights the trajectory of research, illustrating how early chatbot systems paved the way for their current applications in healthcare [31]. This historical context is essential for appreciating the evolution of digital tools and their integration into clinical workflows.

The value of an umbrella review lies in its ability to consolidate and synthesize a diverse range of studies, offering a holistic view of the field. By categorizing the contributions of LLMs and chatbots in radiology, this review reveals emerging themes and their impact on clinical practices [32]. This nuanced perspective not only helps researchers and practitioners understand how these technologies enhance diagnostic accuracy and streamline workflows [35,36] but also highlights their potential to improve patient outcomes [37].

Moreover, the exploration of opportunities and challenges fosters a balanced understanding of the integration of these technologies into clinical settings. By examining potential benefits, such as improved efficiency and accessibility [38], alongside obstacles like data privacy concerns and algorithmic bias [33,34], this review encourages critical dialogue among stakeholders. This balanced approach is vital for navigating the complexities of implementing innovative technologies in healthcare.

Ultimately, this umbrella review serves as a pivotal resource for guiding future research directions and practical implementations. It informs policymakers, healthcare providers, and researchers about current trends and gaps in the literature, facilitating evidence-based decision-making [39]. By highlighting the interconnectedness of findings across studies, this review underscores the need for collaborative efforts to enhance the effectiveness and ethical use of NLP and chatbots in radiology [40], thereby contributing significantly to the advancement of the field.

### 4.2. Discussion: Emerging Recommendations

As the integration of natural language processing (NLP) technologies in radiology continues to progress, it is crucial to establish clear and actionable recommendations to guide their effective implementation. These recommendations can be divided into two main categories: direct and indirect recommendations.

Direct recommendations focus on practical, immediate strategies that can be implemented to optimize the use of NLP technologies in radiology. These include specific guidelines for the adoption of NLP tools in clinical workflows, addressing the technical challenges involved in system integration, and ensuring the appropriate training for radiologists and staff to effectively use these technologies. Direct recommendations are aimed at facilitating the immediate, tangible application of NLP tools in radiology practice.

On the other hand, indirect recommendations address the broader context and systemic factors that impact the long-term success of NLP integration. These encompass the development of interdisciplinary collaborations, establishing standardized processes, addressing ethical concerns, and creating regulatory frameworks to ensure the responsible and effective use of NLP technologies. Indirect recommendations are essential for building a sustainable environment where NLP tools can thrive and be continuously refined to meet evolving clinical needs.

#### 4.2.1. Emerging Direct Recommendations

Direct recommendations focus on concrete, actionable strategies designed to optimize the application of natural language processing (NLP) in clinical environments. These recommendations offer clear guidance on how to implement and improve NLP technologies, ensuring they effectively address the unique needs and challenges of healthcare practice.

By identifying and focusing on concrete steps, such as enhancing structured reporting and addressing data privacy concerns, these recommendations can facilitate immediate improvements in diagnostic accuracy and workflow efficiency. For instance, the enhancement of structured reporting through NLP can lead to standardized documentation practices, which have been shown to reduce discrepancies and improve communication among healthcare providers (Younis et al. [32]; Sacoransky et al. [33]).

Moreover, addressing data privacy concerns is crucial in the implementation of NLP technologies. As these systems often process sensitive patient information, ensuring robust data security measures is vital to maintaining patient trust and compliance with regulations. Recent findings underscore the necessity of developing clear protocols that govern data handling, which can mitigate risks associated with privacy breaches (Keshavarz et al. [34]).

Table 3 outlines these direct recommendations, providing a concise summary below of the actions that can be taken to optimize NLP applications in radiology.

#### 4.2.2. Emerging Indirect Recommendations

Indirect recommendations, in contrast, emphasize broader systemic factors that shape the successful adoption and long-term effectiveness of NLP technologies. These considerations go beyond immediate technical challenges and delve into critical themes such as ethical oversight, interdisciplinary collaboration, and the integration of NLP tools with existing medical devices. By addressing these larger, contextual factors, we can ensure that the implementation of NLP technologies is not only effective but also responsible, ethical, and sustainable in the long run.

Ethical oversight is particularly critical in the realm of NLP, where biases in training data can lead to disparities in clinical outcomes. Establishing comprehensive ethical guidelines is essential to ensure that NLP applications are developed and implemented with fairness and transparency (Dias et al. [45]).

Collaboration across disciplines, including radiology, informatics, and ethics, can foster a more holistic approach to integrating NLP into clinical practice. Interdisciplinary teamwork can lead to the design of more effective solutions that consider the diverse needs of stakeholders involved in patient care (Pons et al. [39]).

Furthermore, ensuring that NLP systems are compatible with existing medical devices can enhance overall workflow efficiency. By facilitating seamless integration, healthcare providers can better leverage these technologies to improve patient outcomes (Gorenstein et al. [36]).

Table 4 presents these indirect recommendations below, highlighting the foundational considerations necessary for fostering an environment conducive to the successful implementation of NLP in radiology. Prioritization of these recommendations is critical to guide efforts effectively, and this responsibility should lie with stakeholders such as regulatory bodies, professional organizations, research institutions, and healthcare providers.

By prioritizing these areas, these actors can ensure that development aligns with clinical needs, ethical standards, and technological advancements. For example, ethical oversight might take precedence as it forms the bedrock for responsible deployment, followed by the need for collaboration and standardization to harmonize innovation and clinical integration. Finally, compatibility with medical devices ensures that NLP seamlessly supports and enhances existing workflows.

Overall, the recommendations outlined above aim to provide a comprehensive framework for the effective integration of NLP technologies in radiology. By distinguishing between direct and indirect recommendations, we can address both the immediate needs of clinical practice and the broader systemic challenges that must be overcome to fully realize the potential of NLP in enhancing diagnostic and patient care.

### 4.3. Integrating Insights: How Indirect Recommendations Complement Direct Approaches in Complementary NLP Implementation with Primary Sources

Indirect recommendations play a critical role in guiding large-scale research initiatives by addressing systemic factors that profoundly affect the effectiveness and long-term sustainability of NLP technologies in clinical settings. Unlike direct recommendations, which focus on the immediate technical applications of NLP, indirect recommendations examine the broader contexts in which these technologies function. By addressing these larger systemic considerations, they have the potential to create a more significant and lasting impact on the adoption and integration of NLP in healthcare.

Firstly, establishing ethical oversight is essential for guiding research that seeks to understand and mitigate potential biases in NLP algorithms. Without a robust ethical framework, research may inadvertently perpetuate existing disparities in healthcare outcomes, undermining the potential benefits of NLP technologies (Dias et al. [45]). By prioritizing ethical considerations, researchers can better assess the societal implications of their work, leading to more equitable solutions that improve patient care across diverse populations.

Secondly, promoting collaboration across disciplines can enhance the depth and breadth of research on NLP applications. Interdisciplinary partnerships can facilitate knowledge exchange among radiologists, data scientists, ethicists, and policymakers, fostering innovative approaches to the challenges posed by NLP integration. This collaborative spirit is vital for developing research agendas that address not only technical aspects but also user needs, regulatory requirements, and clinical workflows (Pons et al. [29]).

Additionally, the standardization of reporting practices in NLP research can significantly enhance the reproducibility and comparability of studies. When researchers adhere to common reporting standards, it enables more effective synthesis of findings across different studies, facilitating meta-analyses and larger systematic reviews. This cohesive research framework can illuminate gaps in knowledge and identify priority areas for further investigation (Davidson et al. [41]).

Moreover, ensuring compatibility of NLP systems with existing medical devices fosters a more seamless integration into clinical workflows. This compatibility can serve as a catalyst for future research, focusing on optimizing human–machine interaction and enhancing overall system performance. By addressing these broader systemic considerations, researchers can create a more conducive environment for the successful implementation of NLP technologies in radiology, ultimately leading to improved diagnostic accuracy and patient outcomes.

Overall, indirect recommendations play a vital role in guiding large-scale research initiatives by addressing ethical, collaborative, and systemic factors that influence the deployment of NLP technologies. While direct recommendations provide actionable steps for immediate improvement, it is the indirect recommendations that offer a more comprehensive framework for ensuring the responsible and sustainable integration of NLP into healthcare systems. By emphasizing these broader contexts, we can create an environment that not only enhances the effectiveness of NLP applications but also fosters equitable patient care across diverse populations.

It is important at this point to integrate this umbrella review with cutting-edge research in areas directly related to the indirect recommendations. This integration not only enriches this review by incorporating the latest advancements but also ensures that the findings are relevant to current practices and future developments in the field. By connecting the insights gained from this umbrella review with the forefront of research, we can better address the systemic and contextual issues that influence the successful implementation of NLP and chatbot technologies in healthcare settings.

Box 2 reports the keyword used in the further analysis.

Box 2. **The proposed composite search keyword.**

*((chatbot[Title/Abstract]) OR (NLP[Title/Abstract]) OR (chatgpt[Title/Abstract]) OR(natural language processing[Title/Abstract]) OR (natural language model[Title/Abstract])) AND (radiology [Title/Abstract]) AND (ethics[Title/Abstract])*


*((chatbot[Title/Abstract]) OR (NLP[Title/Abstract]) OR (chatgpt[Title/Abstract]) OR(natural language processing[Title/Abstract]) OR (natural language model[Title/Abstract])) AND (radiology [Title/Abstract]) AND ((Partnership [Title/abstract]) OR (Synergy [Title/abstract]) OR (Teamwork [Title/abstract]) OR (Cooperation [Title/abstract]) OR (Networking [Title/abstract]) OR (Joint Efforts [Title/abstract]) OR (Interdisciplinary [Title/abstract])OR (Stakeholder Engagement [Title/abstract]) OR (Collaboration Framework [Title/abstract])OR (Shared Goals [Title/abstract]) OR (Cross-functional [Title/abstract]) OR (Collective Impact [Title/abstract])OR (Knowledge Exchange [Title/abstract])OR (Co-creation [Title/abstract]) OR (Alliance [Title/abstract]))*


*((chatbot[Title/Abstract]) OR (NLP[Title/Abstract]) OR (chatgpt[Title/Abstract]) OR(natural language processing[Title/Abstract]) OR (natural language model[Title/Abstract])) AND (radiology [Title/Abstract]) AND ((medical device[Title/Abstract]) OR (standard[Title/Abstract]) OR (regulation[Title/Abstract]))*

#### 4.3.1. Ethical Considerations in NLP/NLP/Chabot Applications in Radiology

*When focusing on the first indirect recommendation* (Table 4), recent studies underscore the transformative potential of artificial intelligence (AI) in radiology, particularly through the use of large language models (LLMs), natural language processing (NLP), and chatbots. However, as these technologies evolve, it is essential to address the critical ethical considerations that arise with their integration into clinical practice. These ethical concerns include issues related to data privacy, algorithmic bias, transparency, and informed consent, all of which play a pivotal role in ensuring that AI tools are used responsibly and equitably in radiology. The complementing ethical analysis, based on the key in Box 2, position 1, yields six results with primary articles available starting from 2022.

These articles collectively stress the importance of transparency, accuracy, and equitable outcomes in AI applications:

*Transparency and Methodology*: Reichenpfader et al. (2023) advocate for clear methodologies in studies using LLMs for information extraction from radiology reports. They argue that transparent research practices are essential for building trust and ensuring the reliability of AI tools in clinical settings [46].

*Linguistic Integrity*: Teixeira da Silva (2024) raises concerns about the use of AI in academic writing, highlighting the need for authors to declare their use of tools like ChatGPT. This call for clarity aims to uphold the integrity of the radiology literature and maintain effective communication among professionals [47].

*Equity and Subgroup Analysis*: Ahluwalia et al. (2023) underscore the ethical imperative of conducting subgroup analyses to identify performance disparities in AI classifiers across different patient demographics. Their findings point to the necessity of addressing these disparities to promote equitable healthcare [48].

*Academic Integrity and AI Risks*: Currie (2023) discusses the ethical challenges associated with AI-generated content in academic settings. The author warns about the potential for misinformation and emphasizes the need for robust standards governing AI use in scientific writing to uphold professional ethics [49].

*Data Ethics and Accountability*: Mithun et al. (2023) present a framework for classifying lung carcinoma reports, focusing on ethical considerations regarding data use and the importance of accountability in NLP applications. They argue that the careful management of patient data is critical to ensuring patient safety and trust in AI [50].

*Integration of AI in Clinical Workflows*: Wilson et al. (2022) explore the potential of AI to enhance workflows in veterinary radiology. However, they highlight the ethical challenges that arise during AI integration, particularly in terms of data management and the implications for clinical decision-making [51].

Table 5 reports the ethical considerations in recent research on large language models, natural language processing, and chatbots in radiology.

#### 4.3.2. The Role of Collaboration in NLP/NLM/Chatbots in Radiology

When focusing on the second indirect recommendation (Table 4), we performed a focused study search.

Using the key indicated in Box 2, position 2, we find 12 studies starting from 2014. After excluding reviews and focusing on the most recent ones, we selected five relevant articles [52,53,54,55,56]. The recent literature showcases diverse examples of such collaborative applications, highlighting the roles that various stakeholders play and how they can work together effectively.

Radiology residency applications: Gordon et al. (2024) [52] address the role of LLMs in radiology residency applications, particularly in generating personal statements. They identify concerns among program directors about the quality and authenticity of AI-generated content, signaling a divide in perspectives on how these tools should be used in the application process. This highlights the need for collaboration among residency program directors, AI experts, and regulatory bodies to establish clear standards and ethical guidelines on the use of LLMs in medical applications. Such collaboration could ensure that AI tools enhance rather than undermine the integrity of the application process.

Natural image captioning: In the work of Reale-Nosei et al. (2024) [53], the focus is on natural image captioning in medical diagnostics. The study highlights how collaboration between computer vision specialists and NLP experts can improve the accuracy and clarity of radiology reports. By working together, these professionals can develop more sophisticated models that not only interpret medical images more effectively but also generate descriptions that are useful for clinicians. This collaborative approach ensures that both the technical and clinical aspects of radiology are addressed, leading to better diagnostic outcomes and enhanced communication within healthcare teams.

Patient-centered care: Fink (2023) [54] explores the role of LLMs in patient-centered care, particularly in fostering communication between patients and radiologists. LLMs can facilitate more accessible and personalized communication, aiding patients in understanding complex medical terminology and enabling more informed consent processes. Collaboration between radiologists, data scientists, and patient care specialists is crucial in ensuring that these technologies are used to improve the patient experience while maintaining high standards of care and privacy. By working together, these professionals can develop tools that align with patient needs and clinical best practices, ensuring that the integration of LLMs does not compromise the quality of care.

Automated Radiology Report Analysis: Wang et al. (2022) [55] present RadText, a system that leverages text analysis to assist radiologists in reviewing and interpreting reports. The authors advocate for collaboration in developing standardized systems that can streamline workflows, reduce errors, and alleviate the workload on radiologists. Collaboration between radiologists, AI developers, and workflow experts is essential in ensuring that these tools are not only technically sound but also practical in real-world clinical settings. By working together, these groups can ensure that automation complements the radiologist’s role, enhancing efficiency without sacrificing diagnostic accuracy.

Framework for EHR annotation: Park et al. (2021) [56] introduce SOCRATex, a framework for annotating unstructured clinical documents. This work highlights the need for collaboration across clinical and technical domains, including radiologists, healthcare IT professionals, and data scientists. By collaborating, these professionals can create standardized frameworks for annotating medical records, improving the accessibility and usability of electronic health records (EHRs). This would help in integrating clinical data across various specialties, including radiology, ultimately enhancing decision-making and patient care.

Collaboration in recent studies: Table 6 provides an overview of the collaborative roles played by different stakeholders in cutting-edge studies within radiology. The table underscores the importance of interdisciplinary collaboration and the ways in which various professionals can contribute to the successful implementation of NLP and AI tools in radiology. Among these we find (but not only) the following:

1. Radiologists: they are central to the application and interpretation of these technologies, ensuring that they align with clinical needs and improve patient outcomes.
2. Data scientists and AI developers: they are essential for the development and fine-tuning of NLP models, ensuring they are accurate, reliable, and suitable for clinical settings.
3. Healthcare IT professionals: they are key in integrating AI and NLP technologies with existing radiology systems and workflows, ensuring seamless adoption and minimizing disruption.
4. Ethicists and regulatory bodies: they are crucial for establishing ethical guidelines and standards to govern the use of AI in radiology, ensuring that these tools are used responsibly and in accordance with legal and professional standards.
5. Patient Advocates: they represent the interests of patients, ensuring that technologies enhance patient care and do not compromise their rights or experiences.

These studies exemplify the need for a holistic, collaborative approach to integrating NLP and AI in radiology. As the field continues to evolve, fostering collaboration among these diverse actors will be essential in realizing the full potential of these transformative technologies.

#### 4.3.3. Integrating Standardization and Medical Devices with NLP/NLM/Chatbots in Radiology

We addressed the last two insights suggested by the third and fourth indirect recommendations (Table 4) acknowledging their close interconnection. We do not anticipate significant progress in medical device (MD) research, as their development has largely focused on national and international guidelines and regulations. This scenario highlights the importance of integrating emerging technologies such as large language models (LLMs), natural language processing (NLP), and chatbots into clinical processes, while also considering the regulatory and standardization challenges that arise.

Using the key indicated in Box 2, position 3, we find 97 studies starting from 1998. After excluding reviews, editorials, and letters and focusing on the most recent ones, we selected six relevant articles [57,58,59,60,61,62]. These studies highlight the emerging applications of large language models (LLMs), natural language processing (NLP), and chatbots in the field of radiology, with a strong emphasis on standardization and the integration of these technologies into clinical workflows. However, it is important to note that none of the studies address the issue of medical devices, leaving the indirect recommendation unresolved.

In the context of advancing technologies in healthcare, standardization plays a crucial role in ensuring consistency, accuracy, and reliability. The following key findings highlight the importance of standardization in various applications of large language models (LLMs), natural language processing (NLP), and related technologies in clinical settings:

*Standard Protocols for LLM Applications* ([57] Lee JE et al.): The study emphasizes the necessity of standardized protocols for the application of large language models (LLMs) in clinical decision-making, particularly in lung cancer staging. Variability in performance across LLMs and human readers underscores the need for consistency.

*Patient-Facing Report Generation* ([58] Tang CC et al.): The study proposes a structured method for generating colloquial versions of radiology reports to enhance patient understanding. The study highlights the importance of standardization in ensuring readability and accuracy across different medical literacy levels.

*Reporting Guidelines for Information Extraction* ([59] Reichenpfader D, Denecke K): The study suggests a standardized reporting guideline for studies on information extraction from clinical texts. This guideline aims to improve comparability and replicability across research efforts in clinical NLP.

*Metrics for Disease Progression Estimation* ([60] Amorrortu R et al.): The study identifies the need for standardized metrics to evaluate approaches for estimating disease progression in lung cancer. A lack of gold standards complicates the comparison of methodologies, indicating a gap that needs addressing.

*Workflow Optimization in Radiology* ([61] Weikert T et al.): The study demonstrates how deep learning tools can standardize workflows in radiology, reducing interpretation time and improving efficiency. Standardization in tool use can enhance consistency in radiological assessments.

Automation of Incidental Findings ([62] Evans CS et al.): The study highlights the importance of developing standardized validation processes for NLP models used in identifying incidental findings in radiology reports. Ensuring standardization in model performance can improve patient safety and follow-up interventions.

Table 7 reports the role of standardization in recent cutting-edge studies.

### 4.4. Synoptic Diagram of Discussion

**Figure 5:**
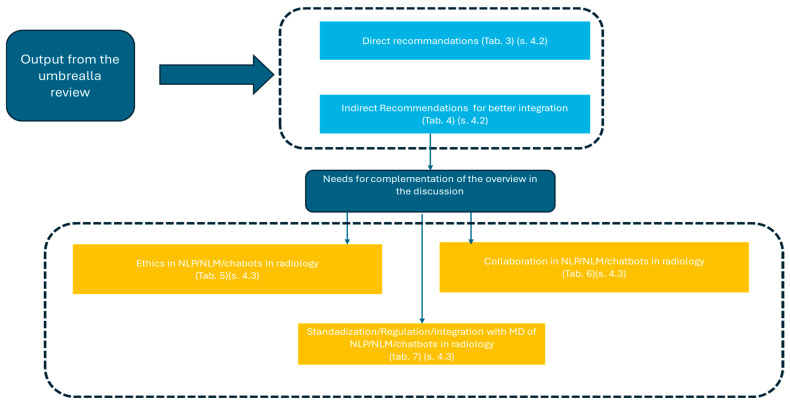
Synoptic diagram presenting an editorial overview of the key tabular highlights in the discussion.

Figure 5 provides a concise schematic overview of the discussion, organized into tabular connections and diagrams that align with the overall progression of the discourse. Based on the limitations and recommendations identified in this review, Figure 5 illustrates the developments in the discussion through a preliminary analysis of key needs. Blocks 1–2 (top right) outline the emerging direct and indirect recommendations summarized in Table 3 and Table 4. Block 3 (below) highlights the need for integration with cutting-edge research. Blocks 4, 5, and 6 (descending and moving from left to right) present, respectively, the complementary findings on ethics (Table 5), collaborations (Table 6), and regulation/standardization (Table 7), noting the absence of studies on medical devices. In the synoptic table is reported with acronym tab. while section with acronym s.

### 4.5. Final Reflection

In summary, ethics represents a critical priority in the adoption of NLP and chatbot technologies in radiology, reflecting broader trends in the emerging field of “algorethics” —the ethics of algorithms [63]. International frameworks have increasingly addressed these issues, setting essential guidelines to ensure ethical standards in AI applications are also applicable here. Organizations such as the World Health Organization (WHO), the European Union (EU), and national regulatory bodies like the U.S. FDA provide comprehensive ethical frameworks applicable to radiology.

The WHO’s global guidelines on AI ethics emphasize human rights, equity, and transparency, urging developers and healthcare systems to prioritize ethical integrity in deploying NLP tools [64]. Similarly, the EU AI Act promotes regulatory alignment across member states by focusing on transparency, fairness, and accountability, shaping practices for the ethical implementation of chatbots and NLP technologies in radiology [65]. These principles guide the development and integration of AI solutions to ensure they support patient care responsibly while mitigating risks.

Specific areas of focus include safeguarding data privacy, fostering equity, and ensuring accountability in algorithmic decision-making processes. For example, FDA guidelines stress transparency and public health safety, ensuring that AI systems, including chatbots and NLP, meet robust ethical and performance standards [66,67]. In parallel, initiatives like the NHS AI Ethics Initiative aim to integrate AI into clinical workflows while managing risks and maintaining public trust [68].

Collectively, these frameworks provide a foundation for prioritizing ethical considerations in the development and deployment of NLP and chatbot technologies in radiology. By aligning technological innovation with established ethical standards, radiology can advance in ways that protect patient rights, foster equity, and promote trust in AI-driven solutions.

### 4.6. Limitations

This review of reviews has certain limitations, primarily related to its focus on the field of NLP/NLM (including chatbots). The goal was to synthesize existing knowledge rather than uncover entirely new perspectives. Consequently, some emerging or less established issues may not be fully addressed. However, this overview also complements the analysis with recent non-review studies aligned with the emerging recommendations from this umbrella review. Specific insights on evolving national and international guidelines are strongly suggested, especially as the discipline continues to develop and analyses increasingly focus on these identified themes.

## 5. Conclusions

This umbrella review has provided a comprehensive assessment of the current landscape of natural language processing (NLP) and natural language models (NLMs), including chatbots, in radiology. These technologies show considerable promise in enhancing clinical decision-making, improving patient engagement, and optimizing communication within radiological workflows. However, challenges remain, particularly the lack of standardized protocols. The absence of consistent guidelines across various applications can compromise the reliability and effectiveness of these tools, preventing their widespread adoption in radiology. To address these challenges, the establishment of clear frameworks for their development and deployment is essential to ensure both safety and consistency in clinical settings.

Another significant gap identified in the literature is the limited exploration of how NLP and NLM technologies can be integrated with medical devices (MDs). As radiology increasingly relies on advanced technologies, understanding how NLP/NLM tools can complement medical devices and enhance diagnostic workflows is critical. Further research is needed to explore these interactions and to develop systems that can seamlessly integrate with MDs, thereby improving the quality of care.

Looking ahead, future research in radiology should focus on several key areas. First, the development of standardized protocols will be crucial for ensuring that NLP and NLM technologies are deployed effectively and consistently. Moreover, understanding the intersection between these technologies and medical devices should be a priority, as this integration could lead to significant improvements in both diagnostic accuracy and patient outcomes. Ethical considerations, including data privacy, informed consent, and the potential for algorithmic bias, also require attention. It is essential that comprehensive ethical guidelines are developed to ensure that these technologies are used responsibly and transparently in clinical settings.

Longitudinal studies are also needed to assess the long-term impact of NLP and NLM technologies on patient outcomes in radiology. These studies will provide valuable insights into their real-world effectiveness, helping refine their applications and ensuring they provide tangible benefits over time.

By addressing these critical areas, future research can help advance the integration of NLP and NLM technologies into radiology, ultimately enhancing diagnostic processes, patient care, and clinical outcomes.

## Figures and Tables

**Table 1:** Key studies on the role of chatbots and NLP in radiology: focus areas and categorization.

Study	Aim	Category	Specific Area
Klug et al. [31]	Explore the role of NLP in improving the patient journey	Clinical NLP	Decision-making, patient journey workflow
Younis et al. [32]	Investigate the applications of ChatGPT in healthcare	AI in healthcare	Various medical sectors including radiology
Sacoransky et al. [33]	Discuss ChatGPT’s role in structured radiology reporting	AI integration	Structured report generation, data extraction
Keshavarz et al. [34]	Explore ChatGPT’s performance and future perspectives in radiology	AI performance review	Diagnosis and clinical decision support
Temperley et al. [35]	Evaluate the current application of ChatGPT in radiology	AI integration	Decision-making, workflow optimization
Gorenstein et al. [36]	Assessment of BERTs’ influence and applications within the radiologic domain	NLP applications	Classification and information extraction
Diab et al. [37]	Overview of recent advances in NLP for breast imaging	NLP in breast imaging	Triage, diagnosis, lesion characterization
Saha et al. [38]	Review NLP applications in radiology reports for breast cancer	NLP in breast cancer	Diagnostic and screening workflows and processes
Pons et al. [39]	Review NLP methods for radiology and their application in clinical practice	NLP applications	Information extraction, clinical application
Linna et al. [40]	Explore recent NLP applications in radiology	NLP applications	Clinical, technical, and quality improvement
Davidson et al. [41]	Assess reporting quality in NLP studies applied to radiology reports	Reporting quality in NLP	Disease classification, diagnostic surveillance
Casey et al. [42]	Evaluate NLP applications in radiology reports	NLP applications	Information extraction from radiology reports
Sorin et al. [43]	Review DL fundamentals in NLP and their application in radiology	DL in NLP	Diagnosis flagging, imaging protocol selection
Blackley et al. [44]	Explore SR technologies for clinical documentation in radiology	SR	Documentation accuracy, turnaround time
Dias et al. [45]	Identify machine learning techniques for assessing physician competence	ML in Medical Competence	Physician assessment through NLP techniques

BERTs: Bidirectional Encoder Representations from Transformers; DL: deep learning; ML: machine learning; NLP: natural language processing; SR: speech recognition.

**Table 2:** Categorization of broad areas: opportunities and limitations in radiology technologies.

Broad Area	Opportunity	Limitation	Key References
Use of ChatGPT in radiology	Enhance radiology reporting by improving accuracy, standardization, decision-making, and workflow optimization, with potential for integration into diagnostic workflows through advanced models	Faces challenges in proficiency, accuracy, data privacy, and medical-specific training, requiring more multicenter studies, diverse datasets, and ethical oversight before widespread clinical adoption	[32,33,34,35]
NLP applied to radiology reports	Offer significant potential to enhance healthcare processes through automated information extraction, improving research and clinical applications	Suboptimal reporting quality, lack of reproducibility, and lack of reproducibility data across studies, necessitating the development of reporting standards and improved collaboration	[36,39,41,42]
NLP in competence assessment	ML techniques offer potential to assess physician competence in real time, enabling more efficient and data-driven evaluations and interventions	More validation research is required to ensure the reliability and effectiveness of ML techniques	[45]
Improvement of clinical practice	NLP models improve clinical practice by automating diagnosis, protocol selection, and follow-up recommendations, enhancing accuracy and efficiency	Effective integration into clinical practice requires healthcare professionals to adapt to new technologies, and more validation is needed to ensure performance across varied clinical environments	[43]
Improvement of diagnostic workflow	Enhance clinical decision-making, patient care, optimize workflows across under-researched areas, and improve specific patient pathway such as breast imaging and care, screening, diagnosis, and patient care through automated data extraction and decision support systems	Lack of data sharing, and the need for thorough evaluation and validation of NLP and AI models hinder their broader clinical adoption and impact on workflow optimization	[31,35,36,37,38,40]

**Table 3:** Emerging direct recommendations.

Recommendation	Description	Key References
Enhance Structured Reporting	Improve the use of NLP to generate structured radiology reports, ensuring consistency and accuracy.	Younis et al. [32]; Sacoransky et al. [33]
Expand NLP Applications Beyond Radiology	Investigate the potential of NLP in various stages of the patient journey, including treatment and aftercare.	Klug et al. [31]
Integration with Advanced AI Models	Explore the integration of NLP with transformer models to enhance reporting efficiency and accuracy.	Temperley et al. [35]
Improve Data Privacy and Security	Address privacy concerns related to NLP-generated data to enhance trust and compliance.	Keshavarz et al. [34]

**Table 4:** Emerging indirect recommendations.

Recommendation	Description	Key References
Ethical Oversight	Establish ethical guidelines for the deployment of NLP to ensure transparency and accountability.	Dias et al. [45]
Collaboration Across Disciplines	Promote interdisciplinary collaboration to enhance the design and implementation of NLP technologies.	Pons et al. [39]
Standardization of Reporting Practices	Develop standardized reporting protocols to facilitate better comparability and reproducibility in NLP research.	Davidson et al. [41]
Integration with Medical Devices	Ensure that NLP systems are compatible with existing medical devices to enhance overall workflow efficiency.	Gorenstein et al. [36]

**Table 5:** Ethical considerations in recent research on large language models, natural lan-guage processing, and chatbots in radiology.

Study	Authors	Year	Ethical Focus	Synthesis of Findings
[46]	Reichenpfader D, Müller H, Denecke K	2023	Transparency in Methodology	Advocates for transparent methodologies in LLM research to ensure reproducibility and trust in AI applications.
[47]	Teixeira da Silva JA	2024	Linguistic Integrity	Calls for accuracy in linguistic descriptors and ethical disclosure of AI tool usage in scientific writing.
[48]	Ahluwalia M et al.	2023	Equity in AI Performance	Emphasizes the need for subgroup analysis to address performance disparities based on demographics.
[49]	Currie GM	2023	Academic Integrity	Discusses the risks of misinformation associated with AI in academic writing and the need for clear standards.
[50]	Mithun S et al.	2023	Data Ethics and Accountability	Focuses on ethical data use and accountability in NLP applications to prevent misinterpretations.
[51]	Wilson DU et al.	2022	Ethical Workflow Integration	Explores the ethical challenges of integrating AI into clinical practice, particularly in data management.

**Table 6:** The role of collaboration in recent studies.

Study	Authors	Year	Potential Actors Involved	Focus on Collaboration	Key Findings
[52]	Gordon EB et al.	2024	Radiology Program Directors, AI Experts, Regulatory Bodies	Residency applications	Concerns over AI-generated statements and the need for standards in residency processes.
[53]	Reale-Nosei G et al.	2024	Radiologists, Computer Vision Experts, NLP Developers	Natural image captioning	Highlights the need for interdisciplinary collaboration in enhancing diagnostic models.
[54]	Fink MA	2023	Radiologists, Data Scientists, Patient Care Specialists	Patient-centered care	Advocates for collaboration to improve communication and patient understanding in radiology.
[55]	Wang S et al.	2022	Radiologists, AI Developers, Workflow Experts	Automated report analysis	Calls for collaboration in developing standardized systems for efficient workflows.
[56]	Park J et al.	2021	Radiologists, Healthcare IT Professionals, Data Scientists	EHR annotation	Demonstrates the importance of collaborative efforts for integrating clinical texts into databases.

**Table 7:** The role of standard and regulation in recent studies.

Study Reference	Authors	Year	Focus	Key Findings
[57]	Lee JE et al.	2024	Lung Cancer Staging Using LLMs	Highlights the need for standardized protocols in LLM applications for clinical decision-making. GPT-4o achieved 74.1% accuracy, indicating variability across LLMs and human readers.
[58]	Tang CC et al.	2024	Generating Colloquial Radiology Reports	Proposes a standardized approach for producing patient-facing reports, enhancing readability and accuracy.
[59]	Reichenpfader D, Denecke K	2024	Reporting Guidelines for Information Extraction	Proposes a guideline to standardize methodologies and outcomes in information extraction from clinical texts.
[60]	Amorrortu R et al.	2023	Disease Progression Estimation	Discusses the need for standard metrics to evaluate disease progression methods using real-world data.
[61]	Weikert T et al.	2023	Deep Learning in Radiology	Demonstrates the potential of deep learning to standardize workflows and reduce time in radiology assessments.
[62]	Evans CS et al.	2023	Identifying Incidental Findings	Highlights the role of NLP in automating findings recognition, suggesting a need for standardized validation processes.

## References

[1] Best D.E., Horii S.C., Bennett W., Thomson B., Snavely D. (1992). Review of the American College of Radiology–National Electrical Manufacturers’ Association standards activity. Comput. Methods Programs Biomed..

[2] Bidgood W.D., Horii S.C., Prior F.W., Van Syckle D.E. (1997). Understanding and using DICOM, the data interchange standard for biomedical imaging. J. Am. Med. Inform. Assoc..

[3] https://www.dicomstandard.org/.

[4] Honeyman J.C. (1999). Information systems integration in radiology. J. Digit. Imaging.

[5] Strickland N.H. (2000). PACS (picture archiving and communication systems): Filmless radiology. Arch. Dis. Child..

[6] Epizitone A., Moyane S.P., Agbehadji I.E. (2023). A Systematic Literature Review of Health Information Systems for Healthcare. Healthcare.

[7] Pirrera A., Giansanti D. (2023). Human-Machine Collaboration in Diagnostics: Exploring the Synergy in Clinical Imaging with Artificial Intelligence. Diagnostics.

[8] https://dicom.nema.org/dicom/dicomwsi/.

[9] Hosny A., Parmar C., Quackenbush J., Schwartz L.H., Aerts H.J.W.L. (2018). Artificial intelligence in radiology. Nat. Rev. Cancer.

[10] Giansanti D., Di Basilio F. (2022). The Artificial Intelligence in DigitalRadiology: Part 1: The Challenges, Acceptance and Consensus. Healthcare.

[11] https://pubmed.ncbi.nlm.nih.gov/?term=%28Artificial+intelligence+%5BTitle%2FAbstract%5D%29+AND+%28radiology%5BTitle%2FAbstract%5D%29&sort=date&size=200.

[12] Maino C., Franco P.N., Talei Franzesi C., Giandola T., Ragusi M., Corso R., Ippolito D. (2023). Role of Imaging in the Management of Patients with SARS-CoV-2 Lung Involvement Admitted to the Emergency Department: A Systematic Review. Diagnostics.

[13] El Naqa I., Li H., Fuhrman J., Hu Q., Gorre N., Chen W., Giger M.L. (2021). Lessons learned in transitioning to AI in the medical imaging of COVID-19. J. Med. Imaging.

[14] Ou P., Wen R., Deng L., Shi L., Liang H., Wang J., Liu C. (2024). Exploring the changing landscape of medical imaging: Insights from highly cited studies before and during the COVID-19 pandemic. Eur. Radiol..

[15] Mese I. (2023). The Impact of Artificial Intelligence on Radiology Education in the Wake of Coronavirus Disease 2019. Korean J. Radiol..

[16] Sun L., Yin C., Xu Q., Zhao W. (2023). Artificial intelligence for healthcare and medical education: A systematic review. Am. J. Transl. Res..

[17] Najjar R. (2023). Redefining Radiology: A Review of Artificial Intelligence Integration in Medical Imaging. Diagnostics.

[18] Clusmann J., Kolbinger F.R., Muti H.S., Carrero Z.I., Eckardt J.N., Laleh N.G., Löffler C.M.L., Schwarzkopf S.C., Unger M., Veldhuizen G.P. (2023). The future landscape of large language models in medicine. Commun. Med..

[19] Aggarwal A., Tam C.C., Wu D., Li X., Qiao S. (2023). Artificial Intelligence-Based Chatbots for Promoting Health Behavioral Changes: Systematic Review. J. Med. Internet Res..

[20] Giansanti D. (2023). The Chatbots Are Invading Us: A Map Point on the Evolution, Applications, Opportunities, and Emerging Problems in the Health Domain. Life.

[21] https://pubmed.ncbi.nlm.nih.gov/?term=%28%28chatbot%5BTitle%2FAbstract%5D%29+OR+%28virtual+assistant%5BTitle%2FAbstract%5D%29+OR+%28agent+based+system%5BTitle%2FAbstract%5D%29+OR%28automated+respond+system%5BTitle%2FAbstract%5D%29%29+AND+%28radiology%5BTitle%2FAbstract%5D%29&sort=date&size=200.

[22] Tudor Car L., Dhinagaran D.A., Kyaw B.M., Kowatsch T., Joty S., Theng Y.L. (2020). Atun R Conversational Agents in Health Care: Scoping Review and Conceptual Analysis. J. Med. Internet Res..

[23] Wang W.T., Tan N., Hanson J.A., Crubaugh C.A., Hara A.K. (2022). Initial Experience with a COVID-19 Screening Chatbot Before Radiology Appointments. J. Digit. Imaging.

[24] Bhayana R. (2024). Chatbots and Large Language Models in Radiology: A Practical Primer for Clinical and Research Applications. Radiology.

[25] Rau A., Rau S., Zoeller D., Fink A., Tran H., Wilpert C., Nattenmueller J., Neubauer J., Bamberg F., Reisert M. (2023). A Context-based Chatbot Surpasses Trained Radiologists and Generic ChatGPT in Following the ACR Appropriateness Guidelines. Radiology.

[26] https://openai.com/chatgpt/overview/.

[27] https://pubmed.ncbi.nlm.nih.gov/?term=ChatGPT.

[28] https://pubmed.ncbi.nlm.nih.gov/?term=%28chatgpt%5BTitle%2FAbstract%5D%29+AND+%28radiology%5BTitle%2FAbstract%5D%29&sort=date&size=200.

[29] https://legacyfileshare.elsevier.com/promis_misc/ANDJ%20Narrative%20Review%20Checklist.pdf.

[30] Lastrucci A., Wandael Y., Barra A., Miele V., Ricci R., Livi L., Lepri G., Gulino R.A., Maccioni G., Giansanti D. (2024). Precision Metrics: A Narrative Review on Unlocking the Power of KPIs in Radiology for Enhanced Precision Medicine. J. Pers. Med..

[31] Klug K., Beckh K., Antweiler D., Chakraborty N., Baldini G., Laue K., Hosch R., Nensa F., Schuler M., Giesselbach S. (2024). From admission to discharge: A systematic review of clinical natural language processing along the patient journey. BMC Med. Inform. Decis. Mak..

[32] Younis H.A., Eisa T.A.E., Nasser M., Sahib T.M., Noor A.A., Alyasiri O.M., Salisu S., Hayder I.M., Younis H.A. (2024). A Systematic Review and Meta-Analysis of Artificial Intelligence Tools in Medicine and Healthcare: Applications, Considerations, Limitations, Motivation and Challenges. Diagnostics.

[33] Sacoransky E., Kwan B.Y.M., Soboleski D. (2024). ChatGPT and assistive AI in structured radiology reporting: A systematic review. Curr. Probl. Diagn. Radiol..

[34] Keshavarz P., Bagherieh S., Nabipoorashrafi S.A., Chalian H., Rahsepar A.A., Kim G.H.J., Hassani C., Raman S.S., Bedayat A. (2024). ChatGPT in radiology: A systematic review of performance, pitfalls, and future perspectives. Diagn. Interv. Imaging.

[35] Temperley H.C., O’Sullivan N.J., Mac Curtain B.M., Corr A., Meaney J.F., E Kelly M., Brennan I. (2024). Current applications and future potential of ChatGPT in radiology: A systematic review. J. Med. Imaging Radiat. Oncol..

[36] Gorenstein L., Konen E., Green M., Klang E. (2024). Bidirectional Encoder Representations from Transformers in Radiology: A Systematic Review of Natural Language Processing Applications. J. Am. Coll. Radiol..

[37] Diab K.M., Deng J., Wu Y., Yesha Y., Collado-Mesa F., Nguyen P. (2023). Natural Language Processing for Breast Imaging: A Systematic Review. Diagnostics.

[38] Saha A., Burns L., Kulkarni A.M. (2023). A scoping review of natural language processing of radiology reports in breast cancer. Front. Oncol..

[39] Pons E., Braun L.M., Hunink M.G., Kors J.A. (2016). Natural Language Processing in Radiology: A Systematic Review. Radiology.

[40] Linna N., Kahn C.E. (2022). Applications of natural language processing in radiology: A systematic review. Int. J. Med. Inform..

[41] Davidson E.M., Poon M.T.C., Casey A., Grivas A., Duma D., Dong H., Suárez-Paniagua V., Grover C., Tobin R., Whalley H. (2021). The reporting quality of natural language processing studies: Systematic review of studies of radiology reports. BMC Med. Imaging.

[42] Casey A., Davidson E., Poon M., Dong H., Duma D., Grivas A., Grover C., Suárez-Paniagua V., Tobin R., Whiteley W. (2021). A systematic review of natural language processing applied to radiology reports. BMC Med. Inform. Decis. Mak..

[43] Sorin V., Barash Y., Konen E., Klang E. (2020). Deep Learning for Natural Language Processing in Radiology-Fundamentals and a Systematic Review. J. Am. Coll. Radiol..

[44] Blackley S.V., Huynh J., Wang L., Korach Z., Zhou L. (2019). Speech recognition for clinical documentation from 1990 to 2018: A systematic review. J. Am. Med. Inform. Assoc..

[45] Dias R.D., Gupta A., Yule S.J. (2019). Using Machine Learning to Assess Physician Competence: A Systematic Review. Acad. Med..

[46] Reichenpfader D., Müller H., Denecke K. (2023). Large language model-based information extraction from free-text radiology reports: A scoping review protocol. BMJ Open.

[47] Teixeira da Silva J.A. (2024). Linguistic precision, and declared use of ChatGPT, needed for radiology literature. Eur. J. Radiol..

[48] Ahluwalia M., Abdalla M., Sanayei J., Seyyed-Kalantari L., Hussain M., Ali A., Fine B. (2023). The Subgroup Imperative: Chest Radiograph Classifier Generalization Gaps in Patient, Setting, and Pathology Subgroups. Radiol. Artif. Intell..

[49] Currie G.M. (2023). Academic integrity and artificial intelligence: Is ChatGPT hype, hero or heresy?. Semin. Nucl. Med..

[50] Mithun S., Jha A.K., Sherkhane U.B., Jaiswar V., Purandare N.C., Dekker A., Puts S., Bermejo I., Rangarajan V., Zegers C.M.L. (2023). Clinical Concept-Based Radiology Reports Classification Pipeline for Lung Carcinoma. J. Digit. Imaging.

[51] Wilson D.U., Bailey M.Q., Craig J. (2022). The role of artificial intelligence in clinical imaging and workflows. Vet. Radiol. Ultrasound.

[52] Gordon E.B., Maxfield C., French R., Fish L.J., Romm J., Barre E., Kinne E., Peterson R., Grimm L.J. (2024). Large Language Model Use in Radiology Residency Applications: Unwelcomed but Inevitable. J. Am. Coll. Radiol..

[53] Reale-Nosei G., Amador-Domínguez E., Serrano E. (2024). From vision to text: A comprehensive review of natural image captioning in medical diagnosis and radiology report generation. Med. Image Anal..

[54] Fink M.A. (2023). Large language models such as ChatGPT and GPT-4 for patient-centered care in radiology. Radiologie.

[55] Wang S., Lin M., Ding Y., Shih G., Lu Z., Peng Y. Radiology Text Analysis System (RadText): Architecture and Evaluation. Proceedings of the 2022 IEEE 10th International Conference on Healthcare Informatics (ICHI).

[56] Park J., You S.C., Jeong E., Weng C., Park D., Roh J., Lee D.Y., Cheong J.Y., Choi J.W., Kang M. (2021). A Framework (SOCRATex) for Hierarchical Annotation of Unstructured Electronic Health Records and Integration into a Standardized Medical Database: Development and Usability Study. JMIR Med. Inform..

[57] Lee J.E., Park K.S., Kim Y.H., Song H.C., Park B., Jeong Y.J. (2024). Lung Cancer Staging Using Chest CT and FDG PET/CT Free-Text Reports: Comparison Among Three ChatGPT Large-Language Models and Six Human Readers of Varying Experience. AJR Am. J. Roentgenol..

[58] Tang C.C., Nagesh S., Fussell D.A., Glavis-Bloom J., Mishra N., Li C., Cortes G., Hill R., Zhao J., Gordon A. (2024). Generating colloquial radiology reports with large language models. J. Am. Med. Inform. Assoc..

[59] Reichenpfader D., Denecke K. (2024). Towards a Reporting Guideline for Studies on Information Extraction from Clinical Texts. Stud. Health Technol. Inform..

[60] Amorrortu R., Garcia M., Zhao Y., El Naqa I., Balagurunathan Y., Chen D.T., Thieu T., Schabath M.B., Rollison D.E. (2023). Overview of approaches to estimate real-world disease progression in lung cancer. JNCI Cancer Spectr..

[61] Weikert T., Litt H.I., Moore W.H., Abed M., Azour L., Noor A.M., Friebe L., Linna N., Yerebakan H.Z., Shinagawa Y. (2023). Reduction in Radiologist Interpretation Time of Serial CT and MR Imaging Findings with Deep Learning Identification of Relevant Priors, Series and Finding Locations. Acad. Radiol..

[62] Evans C.S., Dorris H.D., Kane M.T., Mervak B., Brice J.H., Gray B., Moore C. (2023). A Natural Language Processing and Machine Learning Approach to Identification of Incidental Radiology Findings in Trauma Patients Discharged from the Emergency Department. Ann. Emerg. Med..

[63] Lastrucci A., Pirrera A., Lepri G., Giansanti D. (2024). Algorethics in Healthcare: Balancing Innovation and Integrity in AI Development. Algorithms.

[64] https://www.who.int/news/item/18-01-2024-who-releases-ai-ethics-and-governance-guidance-for-large-multi-modal-models.

[65] https://www.modulos.ai/eu-ai-act/?utm_term=ai%20act%20european%20union&utm_campaign=EU+AI+Act+(December+2023)&utm_source=adwords&utm_medium=ppc&hsa_acc=9558976660&hsa_cam=20858946124&hsa_grp=159677877987&hsa_ad=705319461314&hsa_src=g&hsa_tgt=kwd-2178244031979&hsa_kw=ai%20act%20european%20union&hsa_mt=p&hsa_net=adwords&hsa_ver=3&gad_source=1&gclid=CjwKCAjw5Ky1BhAgEiwA5jGujik2Y5RZXOVwXSvUjE-1RARfMpPgen5q2S7-8FnFFLLIiF052SYAwxoC2oEQAvD_BwE.

[66] https://www.dermatologytimes.com/view/fda-organizations-issue-joint-paper-on-responsible-and-ethical-use-of-artificial-intelligence-in-medical-research.

[67] https://www.pharmacytimes.com/view/fda-issues-paper-on-the-responsible-use-of-artificial-intelligence-in-medical-research.

[68] https://transform.england.nhs.uk/ai-lab/ai-lab-programmes/ethics/#:~:text=The%20AI%20Ethics%20Initiative%20supports,risk%20and%20providing%20ethical%20assurance.

